# Dynamic landscape of chromatin accessibility and transcriptomic changes during differentiation of human embryonic stem cells into dopaminergic neurons

**DOI:** 10.1038/s41598-021-96263-1

**Published:** 2021-08-20

**Authors:** César Meléndez-Ramírez, Raquel Cuevas-Diaz Duran, Tonatiuh Barrios-García, Mayela Giacoman-Lozano, Adolfo López-Ornelas, Jessica Herrera-Gamboa, Enrique Estudillo, Ernesto Soto-Reyes, Iván Velasco, Víctor Treviño

**Affiliations:** 1 Instituto de Fisiología Celular—Neurociencias, Universidad Nacional Autónoma de México, Mexico City, Mexico; 2 Laboratorio de Reprogramación Celular, Instituto Nacional de Neurología y Neurocirugía “Manuel Velasco Suárez”, Mexico City, Mexico; 3 Tecnologico de Monterrey, Escuela de Medicina y Ciencias de la Salud, Monterrey, Mexico; 4 División de Investigación, Hospital Juárez de México, Mexico City, Mexico; 5 Universidad Autónoma Metropolitana—Cuajimalpa, Mexico City, Mexico

**Keywords:** Neurogenesis, Pluripotent stem cells

## Abstract

Chromatin architecture influences transcription by modulating the physical access of regulatory factors to DNA, playing fundamental roles in cell identity. Studies on dopaminergic differentiation have identified coding genes, but the relationship with non-coding genes or chromatin accessibility remains elusive. Using RNA-Seq and ATAC-Seq we profiled differentially expressed transcripts and open chromatin regions during early dopaminergic neuron differentiation. Hierarchical clustering of differentially expressed genes, resulted in 6 groups with unique characteristics. Surprisingly, the abundance of long non-coding RNAs (lncRNAs) was high in the most downregulated transcripts, and depicted positive correlations with target mRNAs. We observed that open chromatin regions decrease upon differentiation. Enrichment analyses of accessibility depict an association between open chromatin regions and specific functional pathways and gene-sets. A bioinformatic search for motifs allowed us to identify transcription factors and structural nuclear proteins that potentially regulate dopaminergic differentiation. Interestingly, we also found changes in protein and mRNA abundance of the CCCTC-binding factor, CTCF, which participates in genome organization and gene expression. Furthermore, assays demonstrated co-localization of CTCF with Polycomb-repressed chromatin marked by H3K27me3 in pluripotent cells, progressively decreasing in neural precursor cells and differentiated neurons. Our work provides a unique resource of transcription factors and regulatory elements, potentially involved in the acquisition of human dopaminergic neuron cell identity.

## Introduction

In eukaryotic cells, DNA is bound to histones forming chromatin, whose structure is dynamic and suffers reversible chemical changes, mainly DNA methylation and histone post-translational modifications. These epigenetic modifications have gained increasing interest since they play fundamental roles in modulating gene expression throughout development, differentiation, and in response to environmental cues^1^. Chromatin modifications alter its packing level, resulting in mainly two distinct environments: ‘open’ accessible regions, also referred to as euchromatin, and ‘closed’ compact regions or heterochromatin. These regions are enriched or depleted in specific histone modifications. Euchromatin is permissive for gene activation, whereas genes in heterochromatin are mainly silenced. Interestingly, regions bound by certain proteins such as CTCF, have a role in maintaining the boundary between heterochromatin and euchromatin^2^. Studies have revealed the role of CTCF in regulating chromatin loops’ formation and, hence, in controlling three-dimensional nuclear landscape to regulate gene expression^3–5^.

Chromatin accessibility is highly dynamic and plays essential roles in establishing and maintaining cellular identity^6^. Euchromatin contributes to the pluripotency of embryonic stem cells (ESCs), whereas heterochromatin regions increase upon differentiation^7,8^. Epigenomic regulation and lineage-specific gene expression orchestrate cellular differentiation; however, the temporal interplay between these mechanisms remains elusive. Several large-scale efforts have characterized transcriptional and epigenetic landscapes of cell lines and tissues^9,10^. Even though these studies have identified putative regulatory elements, none of them focused on dopaminergic differentiation.

Mesencephalic dopaminergic neurons (mDA) are of great medical interest because they are lost in Parkinson’s disease (PD)^11^. Currently, protocols to obtain mDA from ESCs have been described^12^. Differentiated neurons in vitro express several dopaminergic markers, like Tyrosine Hydroxylase (TH), GIRK2, LMX1A, EN1 and FOXA2. These neurons have electrophysiological activities that resemble mDA. Furthermore, ESC derived neurons release dopamine, survive after grafting in parkinsonian non-human mammalian models, and improve the behavioral test performance of these animals, when compared with non-grafted condition^13^. Therefore, differentiation of human ESCs to mDA provides an important model for studying the correlation between the dynamic chromatin accessibility landscape and gene expression changes.

PD is the second most common neurodegenerative disease and its main pathological characteristic is the loss of mDA. Genome-wide association studies (GWAS) have revealed that > 80% of disease-associated SNPs are found within poorly annotated regions^14^, making the identification of causal polymorphisms a difficult task. Disease-associated SNPs in psychiatric and neurological diseases reside in open chromatin regions (OCRs) during neural development^15,16^. Familial PD results from a single mendelian recessive or dominant mutation on one of 11 identified genes, and accounts for roughly 5% of cases^17^. Unlike familial PD, sporadic PD is more complex and results from a combination of environmental and genetic predisposition linked to SNPs.

Here we set out to generate a more detailed genome-wide map of the transcriptional changes (RNA-Seq) and chromatin accessibility (ATAC-Seq) of dopaminergic induction. We integrated epigenomic and transcriptomic profiles to infer the activity of transcription factors (TFs) and DNA regulatory regions such as enhancers and long non-coding RNAs (lncRNAs). Our work provides a unique resource of TFs and regulatory elements, potentially orchestrating human mDA specification.

## Results

### Human ES cells efficiently differentiate into mDA

**Figure 1:**
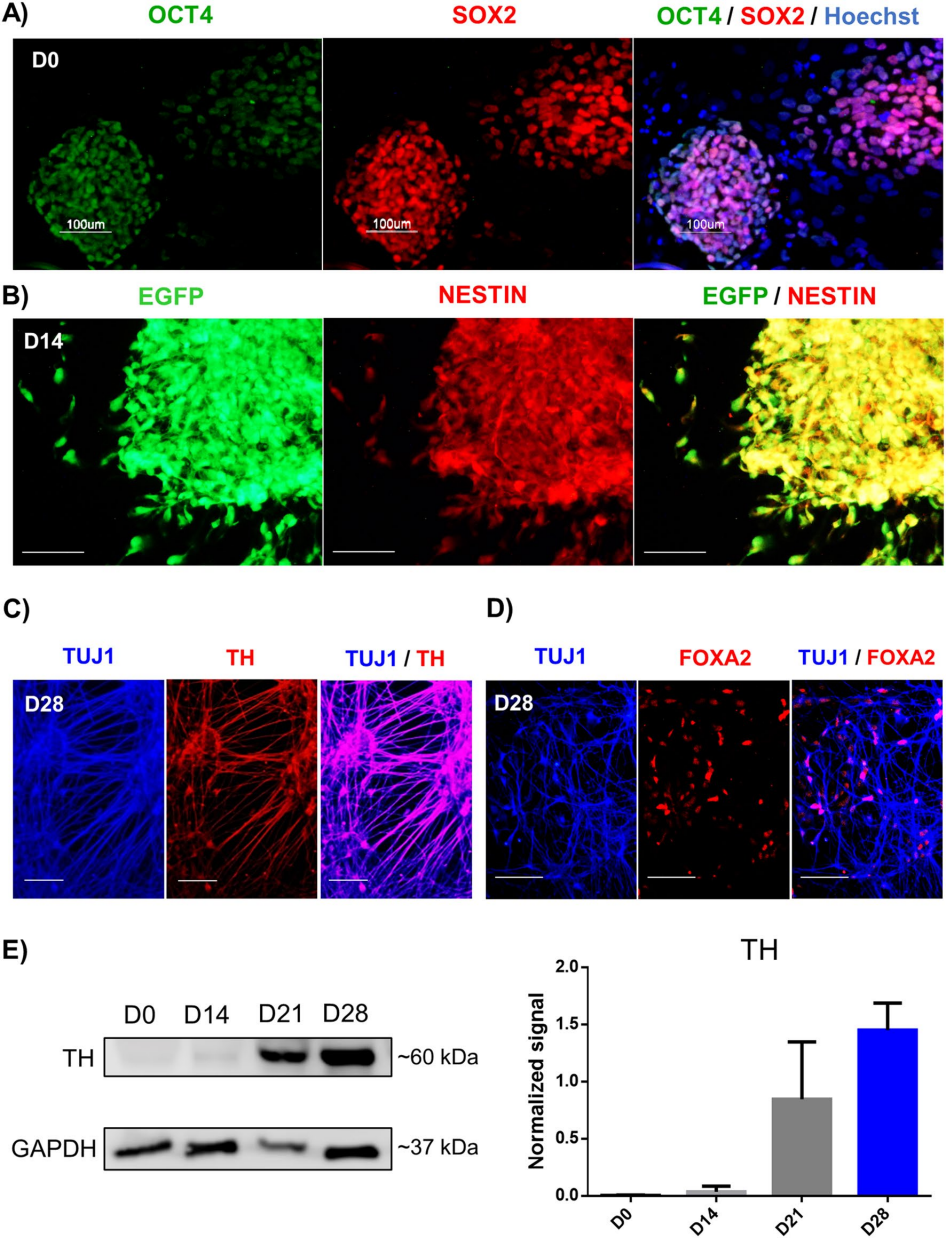
Dopaminergic differentiation of human ESC is progressive, showing three defined stages. (**a**) Fluorescence images depicting immunostaining of pluripotency markers OCT4 and SOX2 in H9-GFP hESC colonies at D0. Nuclei were labeled with Hoechst. (**b**) Immunofluorescence at D14 showing the high proportion of cells positive for NESTIN, a marker of neural precursors and GFP in all cells. (**c**) Staining with the neuronal marker TUJ1 and the dopaminergic marker TH at D28. (**d**) Neurons also express the dopaminergic marker FOXA2, indicating appropriate differentiation. Scale bars, 100 μm. (**e**) Representative Western blot of total cell lysate showing the increase in TH (~ 60 kDa) levels during dopaminergic differentiation. GAPDH was used as a loading control. The graph shows the mean ± SD of two independent experiments.

We differentiated pluripotent human ESC H9-GFP^18^ expressing OCT4 and SOX2 into mDA (Fig. 1a), using a reported floor-plate protocol^12^. NESTIN + neural precursors (NPC) were present at D14 (Fig. 1b). Neurons identified by the BETA-3 TUBULIN (TUJ1) antibody, that co-express TH (Fig. 1c) and FOXA2 (Fig. 1d) are present at D28. It is important to note that at D14, TH is not expressed; this enzyme is detected by immunocytochemistry in 15% of neurons at D24, reaching 50% of TH + neurons at D28 and this percentage increases to 80% at D42^13^. Immunoblot experiments in pluripotent cells and NPC show that they do not express TH, and this enzyme shows increasing levels at D21 and D28 (Fig. 1e). To further characterize the transition from pluripotency to neurons, we performed immunoblots for OCT4, SOX2, and TUJ1. The pluripotency-related transcription factor OCT4 was present only at D0. SOX2 was present in pluripotent cells and was down-regulated at D14, appearing again at D28, consistent with its role in neural differentiation. On the other hand, TUJ1 increased at D14 and D28 (Fig. S1). Thus, the times selected for this study allow a clear distinction between pluripotent cells, neural precursors, and early mDA. Extended periods of differentiation yield mDA that fire action potentials and release DA in vitro at D60 and D70, respectively^13^.

### RNA-Seq reveals transcriptional profiles associated with mDA differentiation

**Figure 2:**
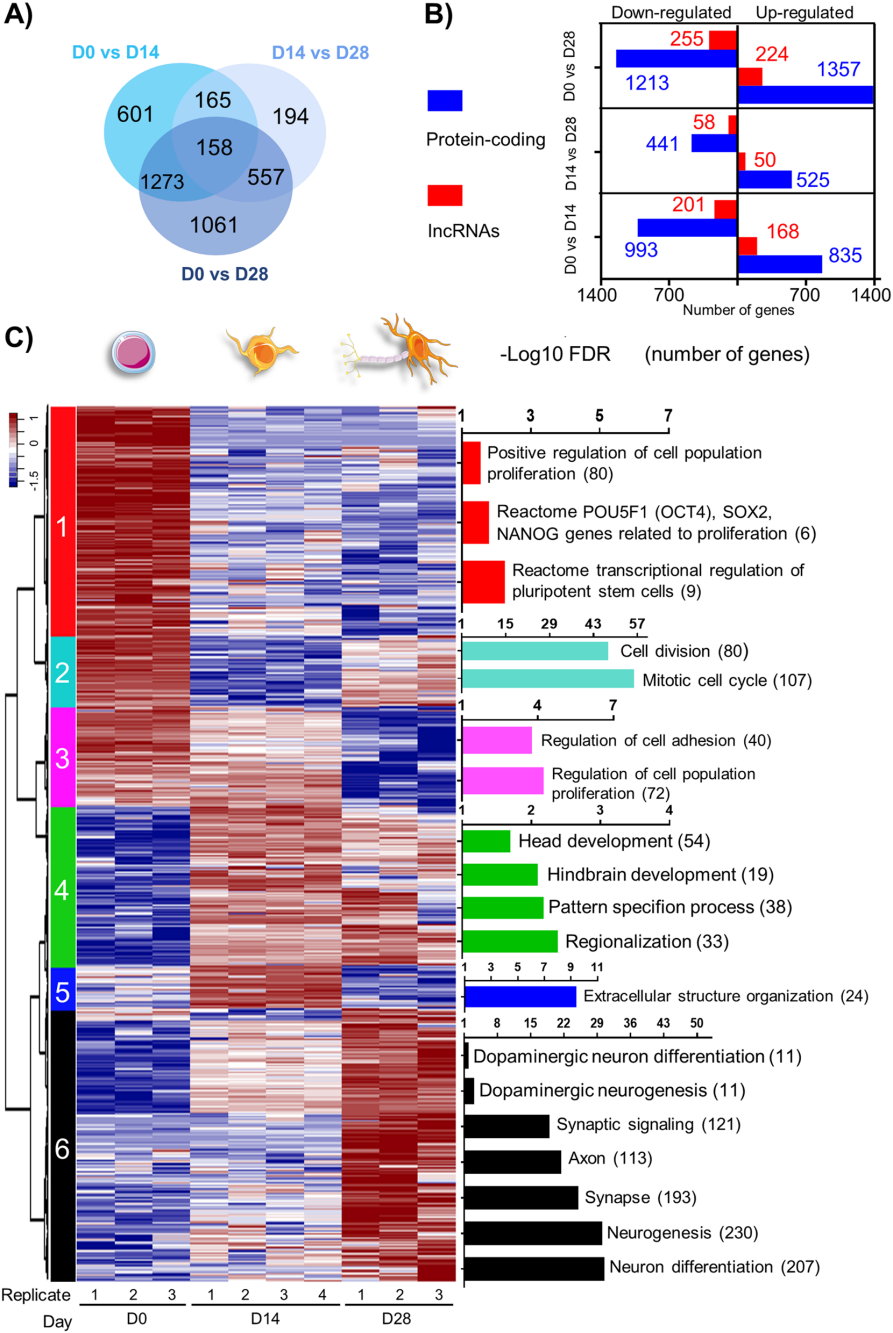
Dopaminergic induction of human ESCs reveals transcriptional dynamic profiles composed of both protein-coding genes and lncRNAs. (**a**) Venn diagram showing the extent of overlap of DEGs found from pairwise comparisons. (**b**) Number of DE protein-coding genes and lncRNAs found in each pairwise comparison. (**c**) Hierarchical clustering analysis displaying temporal expression profiles of all 4009 DEGs compiled from time-point pairwise comparisons. Highly enriched gene-sets and ontologies were identified (hypergeometric test with FDR < 0.05). The enrichment of gene-sets is indicated as − log10(FDR), with the number of genes indicated in parenthesis. Heatmap was built using row z-scores of log2 transformed FPKM gene expression values.

Dopaminergic differentiation can be monitored by mRNA expression: H9 cells at D0 express high levels of *OCT4* and *SOX2*, but not *LMX1A*, *FOXA2* or *TH*, by RT-qPCR; at D14, *LMX1A* and *FOXA2* were detected, and its expression progressively increased. Transcripts for TH were initially detected at D21 and significantly increased afterwards^13^. Here we performed RNA-Seq at D0, D14 and D28 to determine transcriptional profiles underlying early DA induction. The number and category of DEGs found in each comparison are depicted in Fig. 2a,b. A list of 4009 DEGs (3377 protein-coding genes and 632 lncRNAs) was compiled from all comparisons and used for downstream analysis. Gene expression values (FPKM, normalized counts, and batch effect adjusted counts) and statistics of differential expression are included in Supplementary Tables S1 and S2 respectively.

To identify transcriptional patterns, we performed hierarchical clustering of DEGs, resulting in 6 clusters with distinct temporal profiles (Fig. 2c, Supplementary Table S3). Cluster 1 (825 coding genes and 220 lncRNAs) displays downregulation in D14 and D28. Top enriched gene-sets are related to pluripotency and proliferation, consistent with pluripotency loss as cells differentiate. Cluster 2 (309 coding genes and 13 lncRNAs) exhibits significant downregulation in D14, with upregulation at D28; mainly cell cycle gene-sets are enriched. Cluster 3 (410 coding genes and 47 lncRNAs) displays high expression at D0 and D14 and downregulation in D28, with enriched functions related to the regulation of proliferation and cell adhesion. Cluster 4 (581 protein-coding genes and 150 lncRNAs) is upregulated in D14 and decreases its expression in D28. Enriched gene-sets are related to head and hindbrain development, pattern specification, and regionalization. Cluster 5 (163 coding genes and 33 lncRNAs) depicts an upregulation at D14 with a downregulation at D28. Enriched gene-sets include extracellular structure organization. Cluster 6 (1090 coding genes and 168 lncRNAs) displays low levels at D0 and D14 and becomes upregulated at D28. Enriched gene-sets are mainly related to neurogenesis, synapse, axon, and DA neuron differentiation. A heatmap of selected DEGs related to pluripotency and neural DA differentiation is presented in Fig. S3. This analysis confirms that neural lineages are clearly observable from upregulated genes at D14 and maintained at D28 (Cluster 4) or upregulated at D28 (Cluster 6).

### Top significant DEGs are lncRNAs

**Figure 3:**
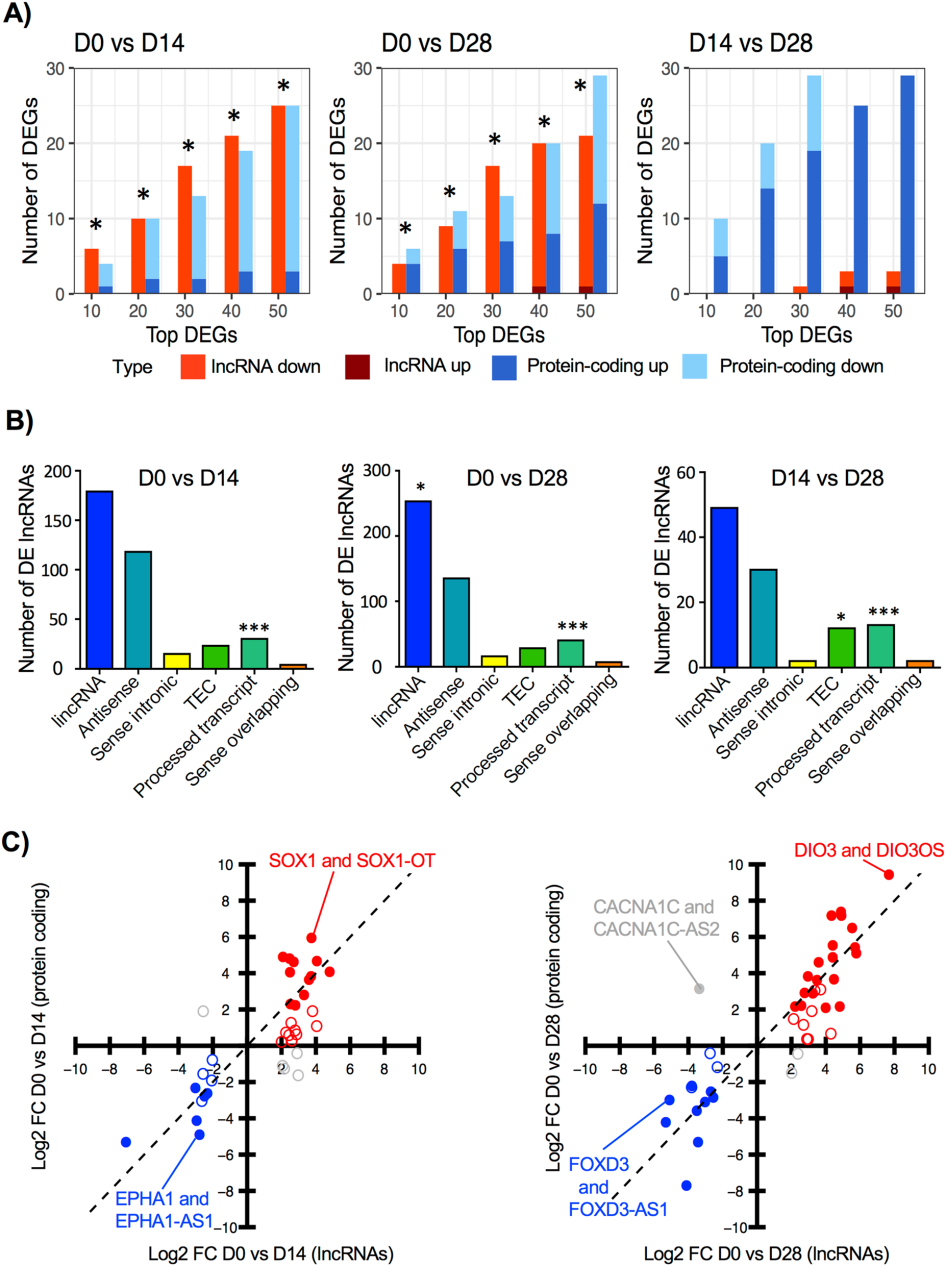
Most significant DEGs found in dopaminergic induction are lncRNAs. (**a**) Number of protein-coding genes and lncRNAs found within the top 10, 20, 30, 40, and 50 DEGs in each pairwise time-point comparison. Protein-coding genes and lncRNAs are depicted in blue and red, respectively with different shades for upregulated (up) or downregulated (down). An asterisk indicates that the *p*-value corresponding to the number of DE lncRNAs was lower than the *p*-value of DE protein-coding genes, indicating higher statistical significance, according to hypergeometric tests. (**b**) Distribution of DE lncRNA gene classes: long intergenic RNAs (lincRNA), antisense, sense intronic, to be experimentally confirmed (TEC), processed transcript, and sense overlapping in pairwise comparisons. *p-value < 0.05, ***p-value < 0.001, according to hypergeometric tests. (**c**) Correlation of expression fold-change (FC) between pairs of DE lncRNAs and their target protein-coding genes. Positive correlations within DE protein-coding and lncRNAs are represented in blue (downregulated) or red (upregulated). Grey circles represent pairs with negative correlation, where pairs of protein-coding and lncRNA move in opposite directions. Filled circles indicate that both lncRNA and protein-coding target gene are DE.

We used the absolute FC to analyze the top ranked DEGs. Overall, lncRNAs are ~ 16% of the DEGs between D0 and D28 (Fig. 2b). Among the top 10 DEGs in neural precursors (D0 vs. D14), 6 are lncRNAs, and only 4 are protein-coding genes (Fig. 3a). Interestingly, the number of lncRNAs within the top (10, 20, 30, 40, and 50) ranked genes is statistically more significant than the number of protein-coding genes (Fig. 3a**,** Supplementary Table S4). A similar pattern is observed when comparing the number of lncRNAs and protein-coding genes between D0 vs. D28. This proportion changed when analyzing the top DEGs between D14 and D28, with a higher significance in the number of protein-coding genes. Almost all lncRNAs within the top 50 DEGs in the comparisons D0 vs. D14 and D0 vs. D28 are downregulated. These results suggest that lncRNAs participate in pluripotency and decrease upon neural differentiation.

We categorized DE lncRNAs according to GENCODE annotation which considers their location relative to protein-coding genes. Using a hypergeometric test, we found that a significant number of DE lncRNAs are classified as processed transcripts and long intergenic non-coding RNA (lincRNA). The high number of lincRNAs suggests potential regulatory functions in *trans* (Fig. 3b). Genes are classified as processed transcripts when their transcripts lack an open reading frame and due to complexity in their structure, they cannot be placed in any other category^19^. Interestingly, the number of DE processed transcripts is significant in all pairwise comparisons. Given the predominance of top DE lncRNAs we searched among all pairwise comparisons and found 96 DE lncRNAs (majority antisense) with known regulatory functions. We found that 56% of DE lncRNAs (54 out of 96) had significant gene expression correlation (Pearson correlation *p*-value < 0.05, Supplementary Table S5) with their target protein-coding genes in samples from all time points. From significant correlations, 91% (49 out of 54) were positive and only 9% (5 out of 54) were negative, suggesting that DE lncRNAs may regulate target protein-coding genes in *cis* and in concordant direction (up- or downregulated). For visualization purposes, we contrasted FCs of DE lncRNAs with their related protein-coding genes (Fig. 3c). Examples of positively correlated genes (either down or upregulated) include *SOX1:SOX1-OT*, *EPHA1: EPHA1-AS1*, *FOXD3:FOXD3-AS1* and *DIO3:DIO3OS*. However, a few pairs, like *CACNA1C*:*CACNA1CA-AS2*, showed a negative correlation.

### Stages of DA induction are characterized by differentially accessible chromatin regions

**Figure 4:**
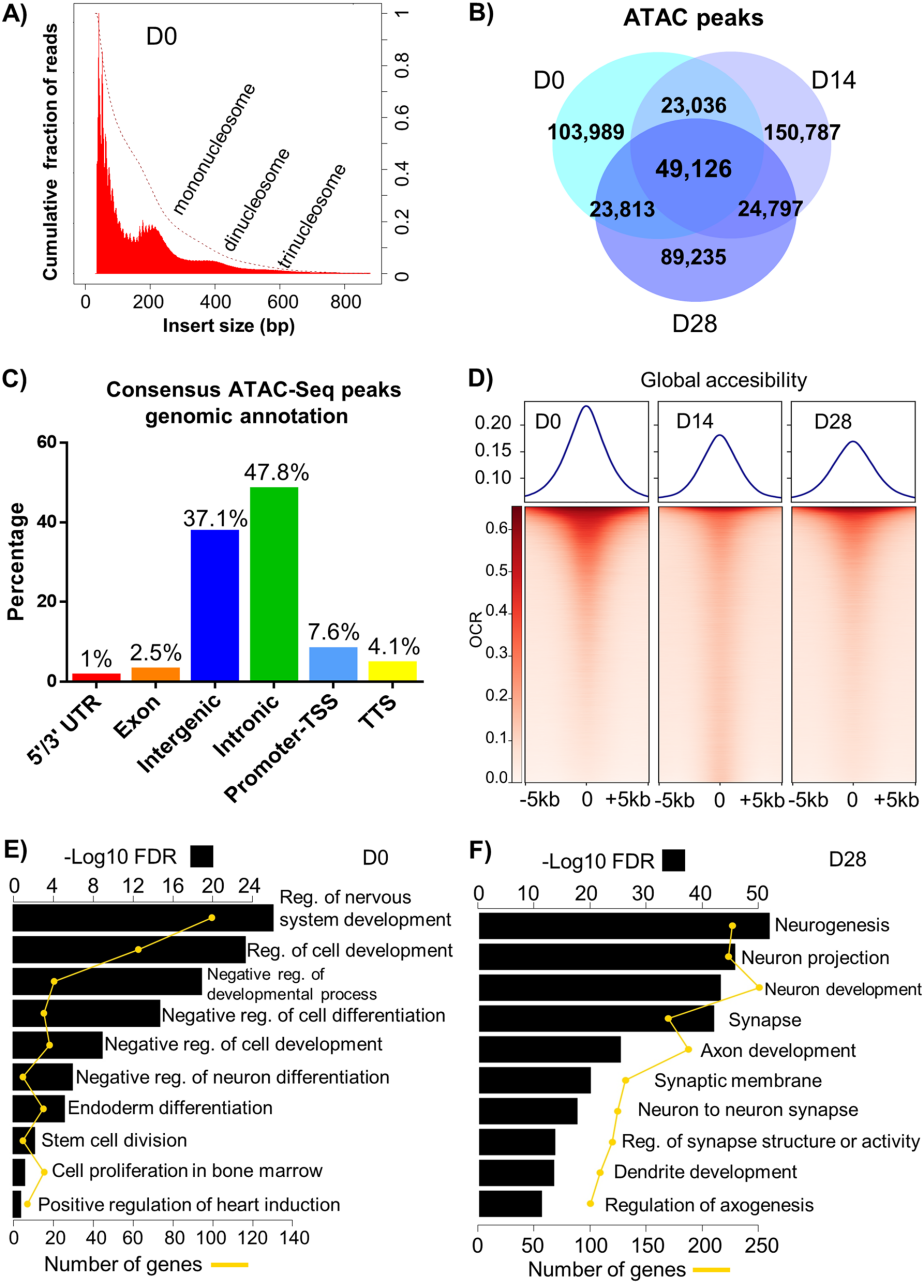
Analysis of chromatin accessibility during dopaminergic induction using ATAC-Seq. (**a**) Fragment size distribution of a representative ATAC-Seq peak sample (D0) provides genome-wide information on chromatin compaction. Fragments smaller than 100 bp represent sequence reads in open chromatin. Reads that span one nucleosome (mononucleosomes) are represented with the peak at 200 bp. Regions of more compact chromatin are indicated by peaks at 440 bps (dinucleosomes) and 600 bps (trinucleosomes). (**b**) Venn diagram representing the number of ATAC-Seq consensus peaks common and unique to open chromatin regions of each time-point. (**c**) Number of consensus ATAC-seq peaks depending on their genomic location. (**d**) Chromatin accessibility at consensus peaks or open chromatin region (OCR) in samples from different induction time-points. Each row represents one consensus peak. Color represents the intensity of normalized chromatin accessibility. Peaks are grouped using k-means clustering. Chromatin accessibility is assessed at regions centered at OCR ± 5 kb. (**e**,**f**) Gene-set enrichment analysis using OCR regions differentially accessible at D0 and D28, respectively. Only OCR regions annotated as promoter-TSS, exon, or intron were used. Selected highly enriched (FDR < 0.05) gene-sets are shown. Black bars depict gene-set enrichment as −log10(FDR) and yellow scale represents number of genes.

We used ATAC-Seq to identify genomic chromatin accessibility depicting active (open) and inactive (closed) chromatin during DA induction. We identified OCRs in samples from D0, D14, and D28 collected simultaneously from the same experiments used for RNA-Seq. Nucleosome packing and an apparent periodicity of approximately 200 bp in insert size distribution plots were evident (Fig. 4a), consistent with previous results^6^. The majority of reads were located in regions less than 100 bp, indicating OCR. Mononucleosome, dinucleosome, and trinucleosome patterns were also observed.

From ATAC-Seq, we identified a total of 464,783 consensus peaks by merging results from all sample time-points. By comparing peak region locations, we found categories of peaks that were either specific to, or shared between time-points. Overall, 103,989 (22%) of the consensus peaks were specific to D0, 150,787 (32%) were D14-specific, and 89,235 (19%) were D28-specific. Only 49,126 (11%) of consensus peaks were shared among all time-points (Fig. 4b). By mapping consensus peaks to genomic regions, we found a higher proportion of peaks in intronic and intergenic regions (Fig. 4c). Due to the fact that we have one sample per time-point, we compared our ATAC-Seq peaks with public data derived from H9 ESCs^20^ to assess the quality of our peak regions. We found that 86% and 93% of ATAC-Seq peak regions in two replicates^20^ overlapped with our D0-specific peak regions.

To analyze global accessibility, we used + /- 5 kb of identified OCRs. Higher chromatin accessibility in D0, compared to D14 and D28 was observed (Fig. 4d), consistent with published results^21^. Additionally, we compared time-point specific OCRs with enhancer regions predicted by GeneHancer^22^ whose database incorporated DNase and H3K27ac assays using H9 pluripotent stem cells. We found that 60% (62,250 out of 103,989) of D0 specific OCRs overlapped GeneHancer annotated regions. In contrast, 31% (46,571 out of 150,787) and 42% (37,700 out of 89,235) of D14 and D28 specific OCR regions respectively, overlapped GeneHancer regions.

To find pathways and gene-sets revealed by OCR specific to different time-points, we tested for statistical differences. We found 2548, 107, and 1236 peaks at D0, D14, and D28, respectively, which were mapped to promoters, exons, or introns to perform a gene-set enrichment analysis (FDR < 0.05). Significant gene-sets enriched in D0-specific peaks are diverse but related to negative regulation of differentiation, stem cell division, and endodermal/mesodermal/ectodermal differentiation (Fig. 4e). In contrast, D28 presented neuronal-related processes, such as neurogenesis, dendrite and axonal development, and synaptic function (Fig. 4f). The list of differentially enriched gene-sets for time-point specific OCRs is included in Supplementary Table S6.

### Temporal patterns of chromatin accessibility are correlated to transcriptomic profiles

**Figure 5:**
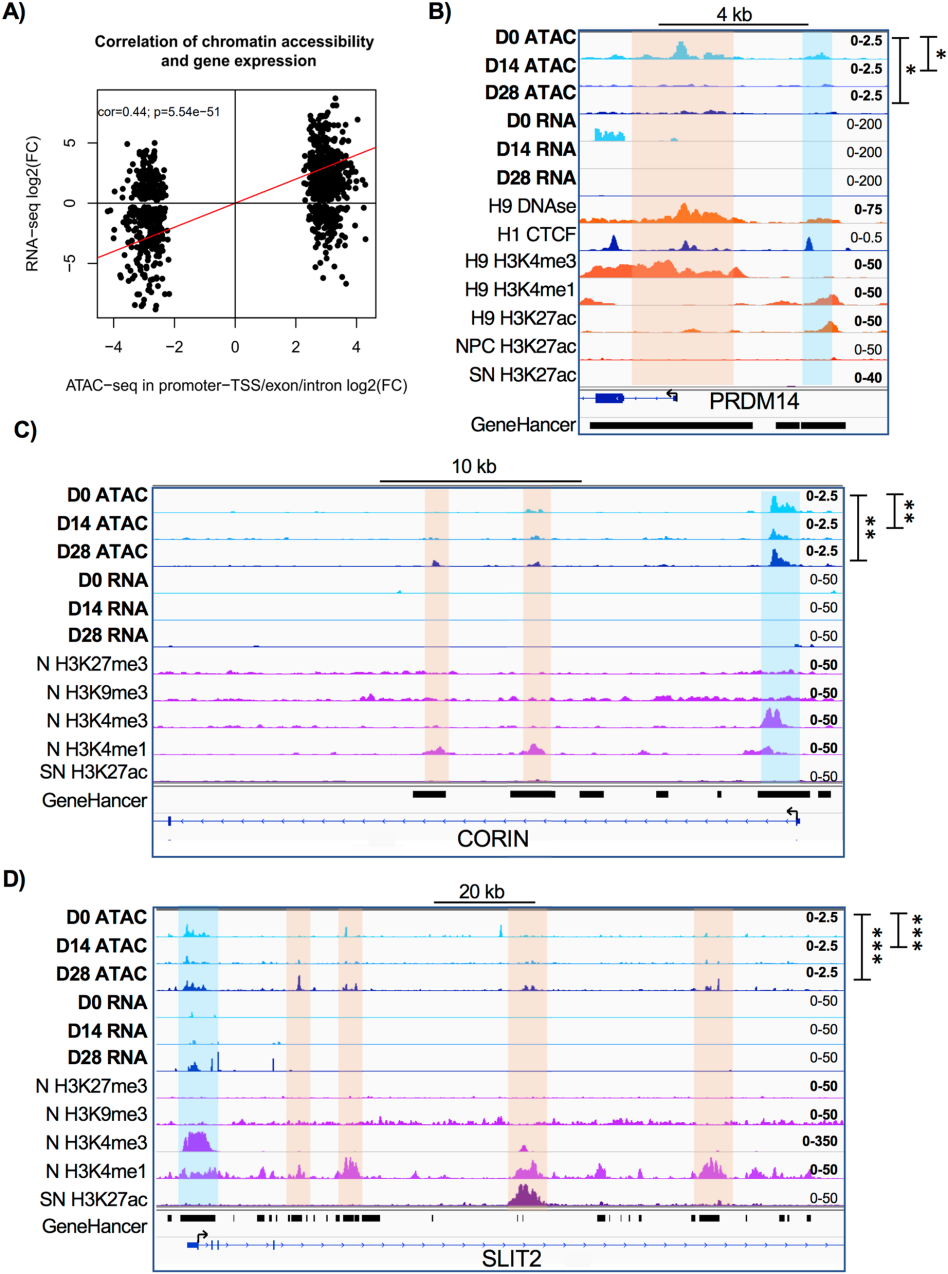
Temporal patterns of chromatin accessibility are correlated to transcriptomic profiles. (**a**) Correlation between gene expression and chromatin accessibility. Consensus peaks annotated as promoter-TSS, exon, and intron were correlated with gene expression. Log2 transformed FC of differential peak accessibility and log2 FC of normalized gene expression counts in the comparison between D0 and D28 were used. (**b**) ATAC-Seq derived chromatin accessibility tracks and RNA-Seq expression changes at the *PRMD14* locus during dopaminergic induction. ENCODE tracks of H9 pluripotent hESC DNAse I, H3K4me3, H3K4me1 and H3K27ac, H1 hESC CTCF, and ESC-derived neural precursors (NPC) H3K27ac are included for comparison. Known and predicted enhancer regions obtained from GeneHancer database are shown. Orange shadow highlights *PRMD14* promoter region and blue region indicates a putative enhancer. Tracks data ranges are shown in the right. (**c**,**d**) Chromatin accessibility and gene expression tracks within *CORIN* and *SLIT2* loci at different dopaminergic induction time-points. ENCODE tracks of ESC-derived neurons (N) H3K27me3, H3K9me3, H3K4me3 and H3K4me1, as well as *substantia nigra* (SN) H3K27ac are included for comparison. Predicted and known enhancer regions derived from GeneHancer database are depicted. Orange zones indicate *CORIN* and *SLIT2* promoter regions. Blue shades highlight putative enhancers. Track data ranges are shown in the right. ATAC-seq tracks labelled by asterisks depict statistical significance obtained by Mann Whitney tests (*p-value < 0.05, **p-value < 0.01, ***p-value < 0.001) using the number of normalized reads in open chromatin regions of gene locus and promoter regions.

To correlate significant differential chromatin accessibility and differential gene expression, we selected consensus ATAC-Seq peaks mapped to promoters, exons, or introns whose *p-*value < 0.05 between D0 and D28. The results show a significant correlation between FC of filtered genes (cor = 0.44, *p*-value = 5.54e−51, Fig. 5a) consistent with previous work^23^.

Coverage plots were used to analyze chromatin accessibility in the promoter regions of DEGs for each cluster depicted in Fig. 2c. A predominant pattern of higher accessibility in D0 compared to D14 and D28 in all clusters was observed (Fig. S4a). Interestingly, we found temporal chromatin accessibility patterns that were similar to transcriptomic profiles. For example, genes in cluster 1 depict a downregulation from D0 to D14 and maintain their lower expression at D28. Genes in cluster 6 display an upregulation from D14 to D28, and a similar pattern is observed in chromatin accessibility. We also compared the chromatin accessibility in promoter regions of DE protein-coding genes and lncRNAs in different clusters. Results show that chromatin accessibility at protein-coding promoter regions is higher at the transcript start site (TSS), whereas slightly shifted at lncRNA TSS (Fig. S4b), potentially due to inaccuracies in lncRNA annotation^24^. In contrast, 500 random non-expressed genes lack signals at TSS (Fig. S4c).

Analyzing individual loci, we observed changes in both chromatin accessibility and expression levels of expected genes at D0 and D28. We complemented our analysis by using public histone modification and DNase I hypersensitivity datasets. Pluripotency genes like *PRDM14* (Fig. 5b) and *NANOG* (not shown) showed a simultaneous significant downregulation of RNA expression and a decrease in OCRs at their promoters. Both loci were also marked by an enrichment of promoter histone mark H3K4me3, the activating histone modification H3K27ac, and DNase I hypersensitivity in H9 undifferentiated hESCs. We also identified a decrease in chromatin accessibility at an upstream region coincident with enhancer histone modification H3K4me1, histone activation modification H3K27ac, and DNase I hypersensitivity. Interestingly, H3K27ac is absent in the same location in hESC-generated NPC. This putative enhancer region is already annotated in GeneHancer database^22^; our results suggest that this region is a pluripotency-related enhancer (Fig. 5b). In contrast, *CORIN* and *SLIT2*, floor-plate markers involved in dopaminergic differentiation^25^ (Fig. 5c,d), depicted a significant increase in gene expression and gained OCRs at both promoter regions and nearby putative enhancer regions, determined by H3K4me1 and H3K4me3 histone modifications in neurons (N) as well as H3K27ac in adult *substantia nigra* (SN). Some of these regions have already been annotated in GeneHancer database.

### Differentially accessible open chromatin regions of D28 coincide with PD-related SNPs

Previous studies have demonstrated that certain disease-associated genetic variants are found within non-coding genomic regions^26,27^. Hence, we compared both consensus and time-point specific OCRs in our experiments with PD-associated single nucleotide polymorphisms (SNPs) derived from GWAS analysis ^28^. Interestingly, significant overlaps were found between PD-related SNPs^28^ and OCRs in consensus peaks and D0 specific peaks only in promoters. Whereas, OCRs specific to mDA (D28) depicted significant overlap with PD-related SNPs only in intronic and intergenic regions. The significancy of overlaps was determined using a hypergeometric statistical test (Supplementary Table S7).

We also assessed the *SNCA* locus, known to be associated with PD-related SNPs^29^, for enrichment in OCR in mDA (D28). SNCA expression and accessibility in OCR overlapping TSS, presumably at the promoter, were invariant through differentiation. In contrast, in the same locus there was an increase in chromatin accessibility at intergenic and intronic OCR by D14 and a higher one at D28 (Fig. S5). Some of these OCR are overlapping with enhancer histone modifications in ESC-derived NPC and N, and importantly, in adult SN. Moreover, the OCR regions identified in D28 in SNCA locus are also annotated in GeneHancer database and appear to be enriched in PD SNPs.

### DEGs are enriched in TF motifs

**Figure 6:**
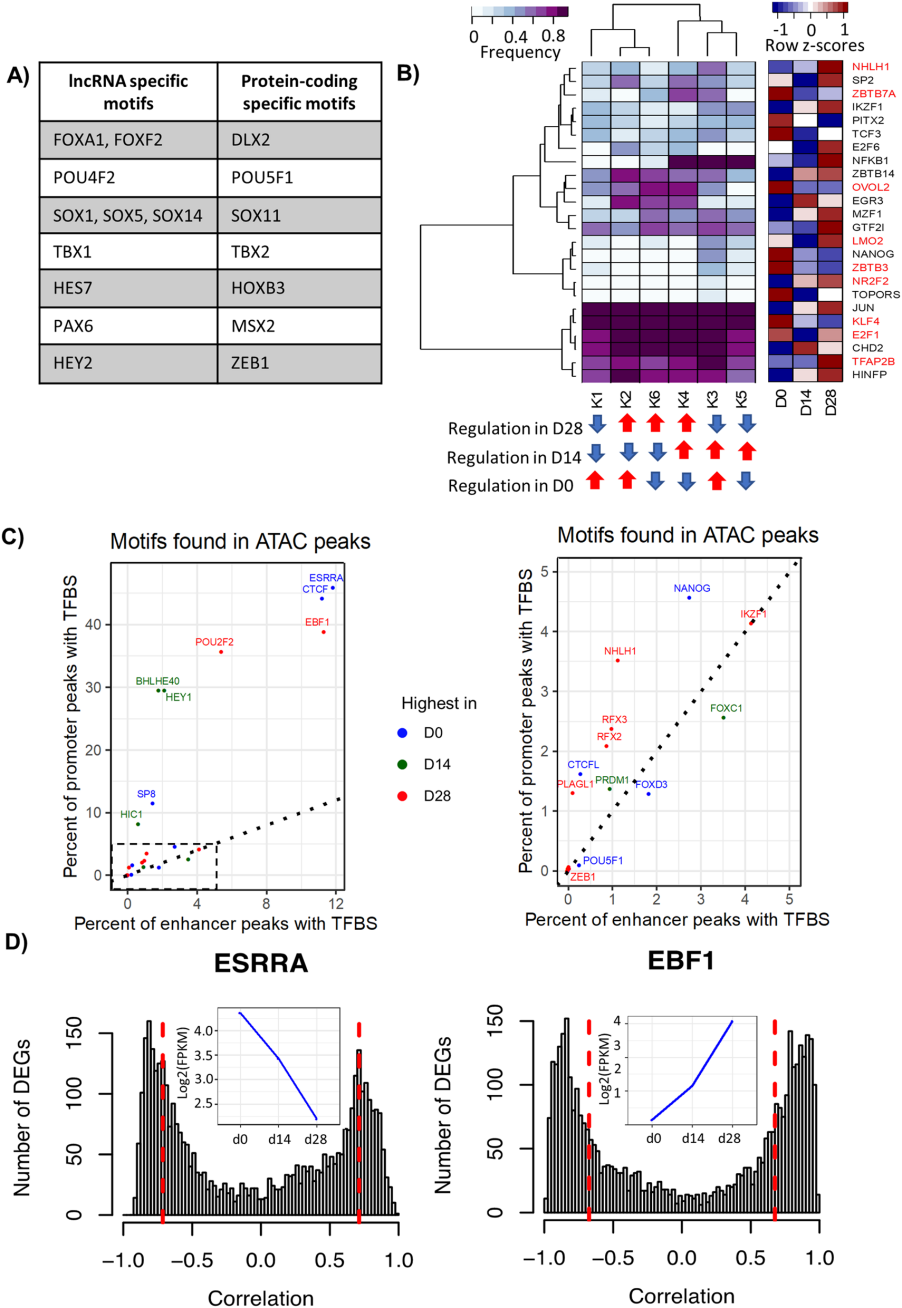
Motif enrichment analysis in DEG promoter regions and open chromatin regions reveals a set of potential TFs involved in dopaminergic differentiation. (**a**) Motifs of TFs found only in lncRNA or in protein-coding gene promoter regions. (**b**) Hierarchical clustering of the frequency of TF binding motifs in promoters of genes from the 6 expression clusters (K) depicted in Fig. 2. White to purple colors represent the density of TF binding in promoter regions of DEGs assigned to expression clusters. The bottom panel highlights the average regulation of genes in each cluster in specific time points. On the right side the RNA expression is shown for D0, D14 and D28. DEGs are labeled with red. (**c**) Enrichment of TF binding motifs in consensus peaks classified as promoters or enhancers. Only motifs of DE TFs are depicted, except for CTCF, which is marginally differentially expressed. Colors indicate the time-point of highest TF gene expression. The right side is a zoom-in of the dashed rectangle in left plot. (**d**) Histogram showing gene expression correlation in all induction time-points between selected highly enriched TFs (ESRRA and EBF1) and DEGs. Dotted red lines represent FDR < 0.05 of Pearson correlation coefficient. The inset blue plots indicate the corresponding TF gene expression at D0, D14 and D28.

From the 3377 DE protein-coding genes, 348 (10.3%) are TFs. We searched for TF binding motifs in promoter regions (± 1 kb up- and downstream) of protein-coding and lncRNA DEGs. For motif search, we used FIMO (FDR < 0.1)^30^ with position weight matrices obtained from ENCODE^31^. Resulting motifs were further filtered to include only those belonging to differentially expressed TFs. We found 65 TFs with significant binding motifs in promoter regions of DE genes, including 48 TFs in protein-coding genes and 49 TFs in lncRNAs. The list of TF motifs found and their metrics are included in Supplementary Table S8. A total of 32 TFs had binding sites in both gene types, whereas motifs of 16 and 17 TFs were found exclusively in protein-coding genes and lncRNAs, respectively. Interestingly, POU5F1, a well-known pluripotency marker depicted binding sites only in protein-coding genes whereas PAX6, a TF involved in the development of neural tissues was specific to lncRNAs. Selected protein-coding and lncRNA-specific TFs with binding motifs are listed in Fig. 6a.

To compare the frequency of TF binding motifs in promoter regions of DE genes from the previously defined expression clusters, we counted the number of genes enriched by each TF binding motif per cluster. This process resulted in a matrix of TFs (rows) and clusters (columns) whose values represent the percentage of genes in each cluster which had a TF binding motif. After filtering out motifs of TFs which were not expressed (FPKM < 1) and TFs whose frequencies remained constant (median absolute deviation < 0.05) in all clusters, we obtained a list of 24 TFs (Fig. 6b, Supplementary Table S8). We observed that TFs such JUN, KLF4, E2F1, and CHD2 have highly occurring motifs in DEGs from all clusters, suggesting global transcriptional regulation. However, there are TFs such as ZBTB7A, EGR3 and NFKB1 with high motif enrichment in specific clusters. In another example, LMO2, and NANOG are TFs with fair enrichment in DEGs of one cluster only. Interestingly, NANOG is upregulated in D0 and depicts binding motifs in DEGs from cluster 3 which have expression upregulation in D0 and D14. Even though not all TFs are DE they might regulate transcription indirectly by interacting with other factors. These results suggest that sets of TFs potentially regulate the coordinated transcriptional changes of genes in clusters while others may have regulatory mechanisms in numerous genes at different time points.

### Motif enrichment analysis in open chromatin regions highlights a preferential location of specific TFs in promoters

To investigate the potential binding of TFs in OCR, we performed a motif search in consensus peak regions. We filtered out motifs with FDR > 0.1. Next, for each TF motif, we calculated its frequency in peaks defined as promoter-TSS and enhancer regions (intergenic, intron, exon, 3’ UTR, and 5’ UTR). Based on ENCODE’s motif matrix, TFs may have more than one motif, thus we selected the most enriched motif representative of a TF. This strategy is intended to identify potentially important regulators of dopaminergic differentiation. We obtained a list of 171 TFs, which were further filtered to contain only TFs in the list of 4009 previously defined DE genes. We found 23 and 18 DE TFs with a higher frequency of binding motifs in promoter or enhancer peaks, respectively (Fig. 6c). The mean frequency of motifs in promoter regions was higher (6.4 ± 13%; range: 0–46%) than in enhancer regions (1.5 ± 13%; range: 0–11.8%). From the TFs with the highest frequency in promoters, 6 were upregulated in D0, 5 in D14, and 12 in D28. Similarly, from the DE TFs with the highest frequency in enhancers, 6 were upregulated in D0, 5 in D14, and 7 in D28. ESRRA and EBF1, upregulated in D0 and D28, respectively were TFs with the highest frequencies in both promoter and enhancer peaks. Even though CTCF is marginally differentially expressed (FC = − 1.65, 1.38, and − 1.16 in D0/D14, D14/D28, and D0/D28, respectively), we added it into the plot because it is highly represented at D0. Supplementary Table S9 lists TFs and their frequencies in promoter and enhancer regions.

### Gene expression correlation between selected TFs and DEGs

The top 8 TFs with motif frequencies annotated to promoter regions were ESRRA, CTCF, EBF1, POU2F2, BHLHE40, HEY1, SP8, and HIC1. Motifs of ESRRA, CTCF, EBF1, and POU2F2 were also the most enriched in enhancer regions. We calculated the gene expression Pearson correlation of these TFs with all 4009 DEGs considering significant correlations at FDR < 0.05. ESRRA, whose expression decreases from D0 to D28, is positively (18%) and negatively (23%) correlated with DEGs, (Fig. 6d). Conversely, EBF1 increases from D0 to D28, and is positively correlated with 33% of DEGs and negatively with 31% (Fig. 6d). Other significant TFs are included in Fig. S6.

### CTCF displays a variable expression during mDA induction

**Figure 7:**
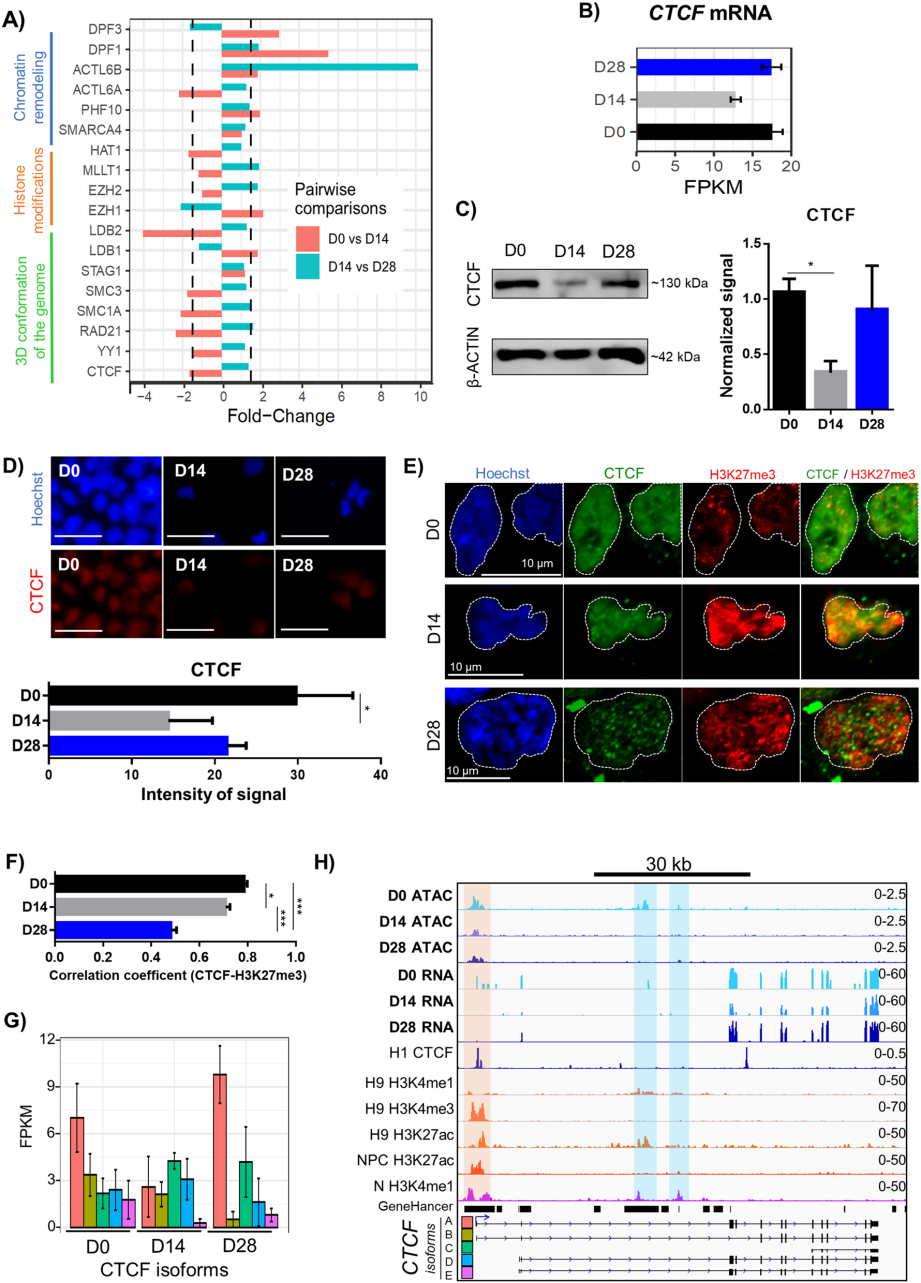
Differential expression of genes that are important for the nuclear landscape and changes in the architectural protein CTCF. (**a**) FC expression of genes involved in chromatin remodeling, histone modifications, and 3D configuration from D0 to D14 and D14 to D28. The dashed vertical lines represent a FC of 1.5. (**b**) Quantification of normalized counts of *CTCF* mRNA levels determined by RNA-Seq during dopaminergic differentiation. (**c**) Representative experiment of CTCF (~ 130 kDa) detection by immunoblot, during dopaminergic induction. β-ACTIN was used as a loading control. On the right side, CTCF quantification at D0, D14 and D28 from 3 independent experiments is shown. * *p* < 0.05. (**d**) Immunostaining of nuclear CTCF during dopaminergic differentiation and its quantification in 5 independent experiments. *p < 0.05. Scale bars, 100 μm. (**e**) Confocal microscope fluorescence images showing nuclear co-localization of CTCF and H3K27me3 through dopaminergic differentiation. (**f**) Correlation coefficient for the co-localization of CTCF with H3K27me3 during differentiation, quantified from confocal images in 30 nuclei per time point. * *p* < 0.05; *** *p* < 0.001. (**g**) Expression of the 5 *CTCF* isoforms at D0, D14 and D28. (**h**) ATAC-Seq chromatin accessibility and RNA-Seq tracks within *CTCF* locus in comparison with ENCODE tracks of H1 CTCF, H9 H3K4me1, H9 H3K4me3, H3K27ac, ESC-derived NPC H3K27ac and ESC-derived N H3K4me1. GeneHancer predicted and known enhancers are included. Five known CTCF isoforms detected by RNA-Seq are also shown. Orange shadow indicates *CTCF* promoter region. Blue shades indicate putative enhancers. Track data ranges are shown in the right.

We identified changes in the expression of important proteins controlling chromatin structure and nuclear architecture (Fig. 7a). The highest FC (9.8) was found in *ACTL6B*, which codes for BAF53B, a subunit of the BAF remodeling complex. Notably, most of the genes associated with 3D chromatin structure show a consistent downregulation from D0 to D14 and an upregulation from D14 to D28. To further analyze chromatin states, we selected CTCF, a major component of the nuclear organization machinery that shows a dynamic expression pattern during DA induction (normalized RNA-Seq counts, Fig. 7b). CTCF protein levels are significantly decreased from D0 to D14, increasing again at D28 (Fig. 7c). A similar CTCF abundance pattern was observed through immunostaining, with a significant decrease between D0 and D14 (Fig. 7d). We then used confocal microscopy to detect the coincidence of CTCF with heterochromatin regions identified by high Hoechst staining (Fig. S7). CTCF is excluded from dense Hoechst regions at D0, and this spatial co-localization is significantly increased at D14 and D28 (Fig. S7). A further analysis was performed to determine the co-localization of CTCF with the Polycomb-repressed chromatin mark H3K27me3, by confocal microscopy. The co-localization of CTCF with this epigenetic modification, which identifies facultative heterochromatin, was assessed by image analysis (Fig. 7e). The correlation coefficient was maximal at D0, significantly decreasing at D14, and more notably at D28 (Fig. 7f). Three-dimensional reconstruction of nuclei of cells labeled with CTCF and H3K27me3 antibodies are shown for D0 (Video S1), D14 (Video S2) and D28 (Video S3).

We noted a variable expression of CTCF isoforms (aliases and transcript IDs in Supplementary Table S10) during DA induction (Fig. 7h). Isoform A has an expression pattern similar to the average of transcripts (gene-level) and protein, depicting a transient decrease in D14 and the highest expression in D28. Isoform B decreases expression with no detectable levels at D28. In contrast, isoforms C and D are upregulated in D14. Isoform E is only expressed in D0 (Fig. 7g**,** Supplementary Table S10). CTCF promoter shows similar levels of chromatin accessibility in our ATAC-Seq samples, to those of H3K27ac reported in pluripotent H9, but also to ESC-derived NPC and N. The latter suggests that CTCF has ubiquitous activity. However, we detected two intronic OCRs within CTCF, previously annotated by GeneHancer. Both regions are markedly accessible at D0, the stage in which CTCF showed the highest transcript and protein levels (Fig. 7b,c), but only one of them overlapped with the H3K27ac mark in H9 hESCs. The region that lacks visible levels of H3K27ac modification in pluripotent cells is also accessible at D28, and interestingly, it displays significant levels of H3K27ac in ESC-derived N. D14, the stage with the lowest CTCF expression, presented only one OCR within the promoter region.

## Discussion

Differentiation of pluripotent human cells to mDA involves morphological changes but also modifications in chromatin structure and RNA expression. Here we adopted a protocol mimicking mDA development^12^ with conditions inducing floor plate precursors prior to dopaminergic neuron specification. We observed the expected downregulation of pluripotent-associated transcripts and the induction of *FOXA2, LMX1A, EN1, EN2, DDC, NR4A2, NEUROG2, NEUROD1, ASCL1, NKX6-1, LMX1B*, and *TH.* Gene-set enrichment analyses were consistent with these processes.

Our characterization of transcriptional changes allowed us to generate a repertoire of DE lncRNAs with potential regulatory functions in pluripotency and mDA commitment. Unexpectedly, there was a high proportion of DE lncRNAs. The majority of DE lncRNAs found were lincRNAs, or antisense relative to the closest protein-coding gene. Notably, most were lincRNAs showing downregulation. Loss-of-function assays in mouse ESCs showed that lincRNAs have key roles in maintaining pluripotency and repressing differentiation programs^32^. Furthermore, lincRNAs identified in ESCs primarily regulate gene expression in *trans*, binding to pluripotency-related TFs, or interacting with chromatin regulatory proteins^32–34^. We found 54 pairs of DE lncRNAs with known regulatory targets which were significantly correlated in all time points. Interestingly, 91% (49 out of 54) showed a positive correlation, suggesting that these lncRNAs participate in positive or negative feedback loops. However, their roles in dopaminergic differentiation remain elusive. LncRNAs such as linc-ROR (lincRNA-ST8SIA3) can reprogram somatic cells to pluripotency^35^. Further studies with simultaneous analysis of TFs, lncRNAs, miRNAs, and epigenetic marks are required to better understand regulatory networks controlling the transitioning from pluripotent stem cells to NPC and mDA.

Previous studies profiled gene expression by microarrays with the same differentiation protocol^12^, and also a comprehensive approach was performed in human DA development with single-cell resolution^36^. However, gene expression is not sufficient for elucidating regulators of cell identity. The integration of ATAC-Seq and the greater resolution of RNA-Seq is a powerful resource to address regulatory heterogeneity between different cellular populations^6^. We found higher levels of global chromatin accessibility in D0 when compared with D14 and D28. These results confirm that ESC have a more open or “loose” chromatin correlating with a more permissive transcriptional state^7^. Differentiation is associated with heterochromatinization and global reorganization of heterochromatin structure, density and position^8^. A clear relationship between chromatin accessibility and gene expression has been demonstrated^37^. We observed a moderate correlation when comparing these parameters, suggesting the participation of other elements in the regulation of gene transcription such as TF binding, DNA methylation, histone tail modifications, miRNAs, and lncRNAs^38,39^.

We identified known and novel TFs potentially orchestrating neurogenesis and dopaminergic specification. We found a higher density of TF motifs in promoter regions compared to enhancer regions, consistent with previous data^40^. By studying DA differentiation in mice, researchers concluded that expression of lncRNAs and accessibility of enhancer regions represent a more accurate molecular signature associated with cell specificity than the expression of protein-coding genes^41^. We found a higher overlap of ATAC-Seq D0-specific OCRs with GeneHancer regulatory elements than with D14 and D28 OCRs suggesting that cell type-specific enhancer regions are potentially involved in dopaminergic differentiation. D14 and D28 OCRs not annotated by GeneHancer are interesting candidates for experimental validation.

Overall, 6 out of 8 DE TFs, whose motifs were most frequently found in OCR, have expression patterns significantly correlated with a considerable number of DE genes. Interestingly, these TFs have diverse profiles with their highest expression in D0, D14, or D28, denoting their roles in pluripotency, neural precursor commitment, or mDA specification. ESRRA and SP8, upregulated in D0, might participate in pluripotency maintenance. ESRRA is a nuclear receptor, but its participation in self-renewal and pluripotency has not been elucidated yet; however, another family member, ESRRB is a crucial regulator of pluripotency in mouse ESCs^42^ and contributes to reprogramming fibroblasts to pluripotency^43^. Recent research revealed a pioneering role of ESRRB in binding silenced enhancers to promote a permissive chromatin state for pluripotent core TF recruitment^44^. Expression profiling studies of nuclear receptors in mouse and human ESCs showed that ESRRB is unique to mice. In contrast, ESRRA is present in both mice and humans^45^, suggesting that ESRRA might subserve biological functions in hESCs similar to those of murine ESRRB.

Motifs of ESRRA, CTCF, and EBF1 were enriched in enhancer regions, suggesting their role in distal regulatory mechanisms. Some of the TFs identified in this study have not been previously implicated in dopaminergic differentiation, but they have a role in neurogenesis. Early B cell factor (EBF1), highly expressed in D28, has been shown to bind to the promoter^46^ and enhancer regions^47^ and may interact with p300/CBP regulating gene transcription^48^. EBF1 is expressed in post-mitotic neurons and has an important role in mouse striatal differentiation^49^. Given that computationally predicted motif binding sites do not always indicate physiological binding of TFs^50^, additional ChIP-seq experiments are needed to address the in vivo validation of TF binding in progenitors and mDA^51^.

Genome-wide association studies (GWAS) have identified associations between SNPs and human pathologies. The majority of identified SNPs lie within non-coding regions including lncRNAs and enhancer regions^26,27^, suggesting that these SNPs mark variations that might impact regulatory functions increasing the risk for PD. Concordantly, we observed that OCRs in differentiated DA neurons overlap significantly with SNPs in non-coding regions. Specifically differentiated DA neurons show that OCRs in *SCNA* overlap with reported PD-related SNPs. Further studies are required to pinpoint the functional effect of enhancer region SNPs with TF binding.

We observed marginal changes in gene expression of several subunits of the chromatin remodeling complex BAF, except for the *ACTL6B* transcript that codes for BAF53B subunit and is significantly upregulated from D14 to D28. Interestingly, cell cycle exit in NPCs is accompanied by a subunit exchange from BAF53A to BAF53B^52^, consistent with our results. To elucidate molecular triggers driving chromatin accessibility changes, we queried expression profiles of diverse chromatin remodelers and selected the architectural protein CTCF, given its importance in brain development^53^. CTCF is ubiquitously expressed in numerous cell types and developmental stages and has been implicated in regulating widespread functions^54^.

The leading hypothesis denoting CTCF’s diverse regulatory functions is by conforming chromatin loops^55^. Notably, we observed a transient decrease at both protein and mRNA levels of CTCF in samples from D14. Beagan et al. found a decreased genome-wide CTCF occupancy in the transition from mouse ES cells to NPCs^53^. They also showed down-regulation in the protein levels of CTCF when comparing mouse pluripotent cells with NPCs, but they did not analyze differentiated neurons; we observed an increase in CTCF when human NPCs progress to differentiated neurons. Heterochromatin regions can be identified by intense Hoechst staining; however, a more specific epigenetic mark for Polycomb-mediated facultative heterochromatin is H3K27me3^56^. Through further assays, we found the lowest co-localization of CTCF with intense Hoechst staining at D0, increasing at D14 and D28. However, the co-localization of CTCF with H3K27me3 in pluripotent cells is highest. At D14, there was a discrete, although significant decrease in this coincidence. In contrast, there was a significant dissociation of CTCF presence with H3K27me3 deposition at D28. Previous work showed that the majority of CTCF sites present in ESC-derived NPC are already present in pluripotent ESC, suggesting that neural commitment is accompanied by a massive loss of CTCF occupancy instead of an extensive CTCF site acquisition or reshuffling^53^. Reduced CTCF presence in heterochromatin at D0 may result from the heterochromatin-free nuclei of ESCs. Increased co-localization of CTCF with heterochromatin might be due to the extensive heterochromatinization that takes place through differentiation^8^ or to CTCF acting as an insulator to prevent excessive repression. Further experimental assays will unveil the role of CTCF in chromatin looping and its changes during DA specification. Our results indicate that CTCF relocates to euchromatin regions in differentiated neurons.

## Conclusion

In summary, we generated genome-wide maps of transcriptional changes and chromatin accessibility and through their integration, we derived a list of TFs and lncRNAs with potential functions in pluripotency maintenance or DA differentiation. We pinpointed important chromatin remodelers and demonstrated the dynamics of CTCF protein and mRNA. Importantly, we highlight the coordinated functions of chromatin accessibility, chromatin remodelers, TFs and lncRNAs to regulate spatiotemporal gene expression. Our work provides a useful resource of TFs and regulatory elements, potentially orchestrating human mDA specification.

## Methods

For detailed protocols, refer to Supplementary Information.

### Dopaminergic differentiation of hESCs

H9 human embryonic stem cells (WA09) of female origin were purchased from WiCell (Madison, WI, USA), transduced with lentiviral vectors to express GFP as described^18^ and cultured until 75% confluency. These GFP-expressing cells are useful for transplantation studies^13^ and behave as its parental H9 hESC. At day zero (D0), cells were considered pluripotent. We followed a dopaminergic induction protocol^12^ with minor modifications^57^. At D14, neural precursors are present, whereas at D28, cells presented a neuronal morphology. Samples were analyzed at D0, D14 and D28, some plates were used for RNA extraction while others for nuclei isolation and chromatin assays.

### Immunostaining

Undifferentiated H9-GFP cells were cultured for 2–3 days. Neural progenitors were re-seeded on induction day 13 or 21 on 24-well plates and fixed the next day. Cells were permeabilized, washed, blocked, and incubated with primary and secondary antibodies. Incubation with Hoechst 33258 (1 ng/ml) was used for nuclear labeling.

### Immunoblot

Standard immunoblot protocols were performed for CTCF and TH. Images were analyzed using ImageJ-Fiji^58^.

### Immunofluorescence analysis of CTCF protein levels

Photographs of CTCF- and Hoechst-stained cells, or confocal images were normalized, and analyzed with ImageJ. Co-localization of CTCF with heterochromatin regions stained with Hoechst was determined by the overlapping area from merged images as reported^56^. Also, the co-localization of CTCF with H3K27me3 was determined as previously described^59^.

### ATAC and RNA sequencing

The assay for transposase-accessible chromatin (ATAC) libraries were prepared on D0, D14, and D28, following the original method^60^. After cell lysis, tagmentation, and adapter incorporation, transposed DNA was purified and quality was assessed. Paired-end 75 cycle sequencing reads were acquired on the Illumina HiSeq 2500 sequencer. A total of 50, 25, and 40 million reads were obtained from D0, D14, and D28 samples respectively.

Total RNA was extracted using TRIzol following manufacturer’s instructions. Samples with RNA integrity number greater than 8 were used. For library construction, Illumina TruSeq RNA Sample Prep Kit (Illumina) were used. Samples were run on MiSeq and HiSeq 2500 Illumina sequencers using paired-end 75 cycle mode. A total of 80.4 million reads were obtained and distributed among 3, 4, and 3 replicates from D0, D14, and D28 time points, respectively.

### ATAC-Seq pre-processing

After removing adaptors using cutadapt^61^ and trimmomatic^62^ we obtained 36–126 bp paired-end ATAC-Seq reads for each time-point samples. The quality of trimmed sequencing datasets was verified using FASTQC^63^. We followed the ENCODE ATAC-Seq pipeline (http://www.encodeproject.org/atac-seq/) to process samples. Paired-end reads were aligned to the human reference genome (GRCh38/hg38) using Bowtie2 with parameters ‘-X 2000 -k 5’^64^. Next, we used SAMTools^65^ to remove unmapped reads, fragments with unmapped mates, non-primary alignment reads, and reads failing platform quality checks. Low quality reads (MAPQ < 30) and reads which mapped to mitochondrial DNA were also excluded. Optical and PCR duplicates were also removed using Picard tools (http://broadinstitute.github.io/picard). To accurately locate the center of each transposon-binding event, the remaining reads were offset by + 4 and − 5 for positive and negative strands respectively^66^. To evaluate the expected periodicity of DNA winding around nucleosomes, we obtained insert size histograms using Picard tools (Fig. 4a). ATAC-Seq peak regions were called for each sample using MACS2 with parameters –shift 75 –extsize 150 –nomodel –keep-dup all –call-summits^67^. Resulting peaks which overlapped with ENCODE blacklisted regions were filtered out^68^.

### Analysis of differential chromatin accessibility

A consensus set of 464,783 unique peaks was generated merging peaks within 100 bp. Exclusive peaks were obtained using BEDTools^69^. Reads in peaks were counted using HTSeq^70^ and tested for differential accessibility using DESeq2^71^ considering |log_2_(fold-change)|> 1.5 and *p*-value < 0.05. Promoter-TSS was defined as 1 kb up- plus 100 bp downstream of the transcript start site (TSS). HOMER annotatePeaks function was used^72^, to associate peaks to genes (GENCODE v29) or genomic regions (intergenic, promoter-TSS, exon, intron, 5’ UTR, 3’ UTR, and TTS). We used Mann Whitney tests to compare the number of normalized reads found in open chromatin regions (OCR) of specific loci between ATAC-Seq samples (D0 vs D14 and D0 vs D28). Asterisks indicate statistical significance (*p*-value < 0.05) in IGV tracks (Fig. 5b–d).

### Gene-set enrichment analysis

The hypergeometric statistical test in R (https://cran.r-project.org/) and a subset of gene-sets from MSigDB^73^ combined with Wikipathways 2019^74,75^ were used. Gene-sets were considered significantly enriched with FDR < 0.05.

### Public datasets

For comparison, we used public datasets for CTCF ChIP-seq, DNase I hypersensitivity, H3K27ac, H3K27me3, H3K4me1, H3Kme3 for H1, H9, NPC, N, LUHMES, and substantia nigra cells (SN) from Gene Expression Omnibus accession numbers indicated in Supplemental information. We used GeneHancer database from GeneCards Suite v4.14^22^ for comparison of enhancer regions and predicted target genes.

### Transcription factor binding analysis

We performed a motif search using position weight matrices from the ENCODE TF ChIP-seq datasets^31^. Promoter regions of differentially expressed (DE) genes (DEGs) (1 kb up- and downstream of the TSS) and open chromatin regions (OCRs, common and differentially accessible) were scanned for motif occurrences using FIMO^30^ with FDR < 0.1. The most frequently occurring DE TFs were correlated with DEGs using the Pearson coefficient. Only correlations with FDR < 0.05 were considered significant.

### Gene expression analysis

Standard quality procedures were performed using cutadapt^61^, trimmomatic^62^, and FASTQC^63^. Reads were mapped to hg38 (https://www.gencodegenes.org/) using a previously described pipeline^76^. Assembly of mapped reads was performed with Cufflinks v2.2.1^77^ and values of Fragments Per Kilobase of transcript per million Mapped reads (FPKM) were calculated for all annotated genes and transcripts (GENCODE v29, https://www.gencodegenes.org/). We used HTSeq^70^ to calculate read counts for annotated genes and transcripts. We adjusted batch effect (Fig. S2) and performed pairwise comparison of read counts implementing DESeq2^71^. Genes were labelled as differentially expressed (DEG) if at least one of the replicates in the comparison had FPKM $\ge$ 1, and normalized count FC > 4 with an FDR < 0.05. Temporal gene expression profiles of DEGs were obtained using hierarchical clustering. DEGs in each cluster were used for gene-set enrichment analysis.

## Supplementary Information

Supplementary Information 1.

Supplementary Information 2.

Supplementary Information 3.

Supplementary Information 4.

Supplementary Information 5.

Supplementary Information 6.

Supplementary Information 7.

Supplementary Information 8.

Supplementary Information 9.

Supplementary Information 10.

Supplementary Video 1.

Supplementary Video 2.

Supplementary Video 3.

Supplementary Information 11.

## Data Availability

Raw sequencing datasets and processed files have been deposited in NCBI Gene Expression Omnibus (GEO) (http://www.ncbi.nlm.nih.gov/geo) under accession GSE153005.

## References

[1] Berger SL (2007). The complex language of chromatin regulation during transcription. Nature.

[2] Ong C, Corces VG (2009). Interactions: A common evolutionary theme. J. Biol..

[3] Fudenberg G (2016). Formation of chromosomal domains by loop extrusion. Cell Rep..

[4] Haarhuis JHI (2017). The cohesin release factor WAPL restricts chromatin loop extension. Cell.

[5] Nora EP (2017). Targeted degradation of CTCF decouples local insulation of chromosome domains from genomic compartmentalization. Cell.

[6] Buenrostro JD (2018). Integrated single-cell analysis maps the continuous regulatory landscape of human hematopoietic differentiation. Cell.

[7] Efroni S (2008). Global transcription in pluripotent embryonic stem cells. Cell Stem Cell.

[8] Meshorer E (2006). Hyperdynamic plasticity of chromatin proteins in pluripotent embryonic stem cells. Dev. Cell.

[9] Roadmap Epigenomics Consortium (2015). Integrative analysis of 111 reference human epigenomes. Nature.

[10] Thurman RE (2012). The accessible chromatin landscape of the human genome. Nature.

[11] Axelsen TM, Woldbye DPD (2018). Gene therapy for Parkinson’s disease, an update. J. Parkinsons. Dis..

[12] Kriks S (2011). Dopamine neurons derived from human ES cells efficiently engraft in animal models of Parkinson’s disease. Nature.

[13] López-Ornelas A (2020). Human embryonic stem cells-derived dopaminergic neurons transplanted in parkinsonian monkeys recover dopamine levels and motor behavior. BioRxiv.

[14] Buniello A (2019). The NHGRI-EBI GWAS Catalog of published genome-wide association studies, targeted arrays and summary statistics 2019. Nucleic Acids Res..

[15] Forrest MP (2017). Open chromatin profiling in hiPSC-derived neurons prioritizes functional noncoding psychiatric risk variants and highlights neurodevelopmental loci. Cell Stem Cell.

[16] Nott A (2019). Brain cell type–specific enhancer–promoter interactome maps and disease-risk association. Science.

[17] Hatano T, Kubo SI, Sato S, Hattori N (2009). Pathogenesis of familial Parkinson’s disease: New insights based on monogenic forms of Parkinson’s disease. J. Neurochem..

[18] Domingo-Reines J (2017). Hoxa9 and EGFP reporter expression in human Embryonic Stem Cells (hESC) as useful tools for studying human development. Stem Cell Res..

[19] ENCODE (2019). 6 Non-coding RNA characterization. Nature.

[20] Liu Q (2017). Genome-wide temporal profiling of transcriptome and open chromatin of early cardiomyocyte differentiation derived from hiPSCs and hESCs. Circ. Res..

[21] Gaspar-Maia A (2009). Chd1 regulates open chromatin and pluripotency of embryonic stem cells. Nature.

[22] Fishilevich S (2017). GeneHancer: Genome-wide integration of enhancers and target genes in GeneCards. Database.

[23] de la Torre-Ubieta L (2018). The dynamic landscape of open chromatin during human cortical neurogenesis. Cell.

[24] Uszczynska-Ratajczak B, Lagarde J, Frankish A, Guigó R, Johnson R (2018). Towards a complete map of the human long non-coding RNA transcriptome. Nat. Rev. Genet..

[25] Yan CH, Levesque M, Claxton S, Johnson RL, Ang SL (2011). Lmx1a and Lmx1b function cooperatively to regulate proliferation, specification, and differentiation of midbrain dopaminergic progenitors. J. Neurosci..

[26] Hindorff LA (2009). Potential etiologic and functional implications of genome-wide association loci for human diseases and traits. Proc. Natl. Acad. Sci. U. S. A..

[27] Ward LD, Kellis M (2012). Interpreting noncoding genetic variation in complex traits and human disease. Nat. Biotechnol..

[28] Nalls MA (2019). Identification of novel risk loci, causal insights, and heritable risk for Parkinson’s disease: A meta-analysis of genome-wide association studies. Lancet Neurol..

[29] Pierce SE, Tyson T, Booms A, Prahl J, Coetzee GA (2018). Parkinson’s disease genetic risk in a midbrain neuronal cell line. Neurobiol. Dis..

[30] Grant CE, Bailey TL, Noble WS (2011). FIMO: Scanning for occurrences of a given motif. Bioinformatics.

[31] Kheradpour P, Kellis M (2014). Systematic discovery and characterization of regulatory motifs in ENCODE TF binding experiments. Nucleic Acids Res..

[32] Guttman M (2011). LincRNAs act in the circuitry controlling pluripotency and differentiation. Nature.

[33] Nagano T (2008). The Air noncoding RNA epigenetically silences transcription by targeting G9a to chromatin. Science.

[34] Khalil AM (2009). Many human large intergenic noncoding RNAs associate with chromatin-modifying complexes and affect gene expression. Proc. Natl. Acad. Sci. U. S. A..

[35] Loewer S (2010). Large intergenic non-coding RNA-RoR modulates reprogramming of human induced pluripotent stem cells. Nat. Genet..

[36] La Manno G (2016). Molecular diversity of midbrain development in mouse, human, and stem cells. Cell.

[37] Frank CL (2015). Regulation of chromatin accessibility and Zic binding at enhancers in the developing cerebellum. Nat. Neurosci..

[38] Heintzman ND, Ren B (2007). The gateway to transcription: Identifying, characterizing and understanding promoters in the eukaryotic genome. Cell. Mol. Life Sci..

[39] Natarajan A, Yardimci GG, Sheffield NC, Crawford GE, Ohler U (2012). Predicting cell-type-specific gene expression from regions of open chromatin. Genome Res..

[40] Dao LTM (2017). Genome-wide characterization of mammalian promoters with distal enhancer functions. Nat. Genet..

[41] Gendron J (2019). Long non-coding RNA repertoire and open chromatin regions constitute midbrain dopaminergic neuron—specific molecular signatures. Sci. Rep..

[42] Martello G (2012). Esrrb is a pivotal target of the Gsk3/Tcf3 axis regulating embryonic stem cell self-renewal. Cell Stem Cell.

[43] Feng B (2009). Reprogramming of fibroblasts into induced pluripotent stem cells with orphan nuclear receptor Esrrb. Nat. Cell Biol..

[44] Adachi K (2018). Esrrb unlocks silenced enhancers for reprogramming to naive pluripotency. Cell Stem Cell.

[45] Xie CQ (2009). Expression profiling of nuclear receptors in human and mouse embryonic stem cells. Mol. Endocrinol..

[46] Treiber T (2010). Early B cell factor 1 regulates B cell gene networks by activation, repression, and transcription- independent poising of chromatin. Immunity.

[47] Lin YC (2010). A global network of transcription factors, involving E2A, EBF1 and Foxo1, that orchestrates B cell fate. Nat. Immunol..

[48] Zhao F, McCarrick-Walmsley R, Åkerblad P, Sigvardsson M, Kadesch T (2003). Inhibition of p300/CBP by early B-Cell factor. Mol. Cell. Biol..

[49] Garel S, Marín F, Grosschedl R, Charnay P (1999). Ebf1 controls early cell differentiation in the embryonic striatum. Development.

[50] Wasserman WW, Sandelin A (2004). Applied bioinformatics for the identification of regulatory elements. Nat. Rev. Genet..

[51] Johnson DS, Mortazavi A, Myers RM, Wold B (2007). Genome-wide mapping of in vivo protein-DNA interactions. Science.

[52] Yoo AS, Staahl BT, Chen L, Crabtree GR (2009). MicroRNA-mediated switching of chromatin-remodelling complexes in neural development. Nature.

[53] Beagan JA (2017). YY1 and CTCF orchestrate a 3D chromatin looping switch during early neural lineage commitment. Genome Res..

[54] Ong CT, Corces VG (2014). CTCF: An architectural protein bridging genome topology and function. Nat. Rev. Genet..

[55] Phillips JE, Corces VGCTCF (2009). Master weaver of the genome. Cell.

[56] Linhoff MW, Garg SK, Mandel G (2015). A high-resolution imaging approach to investigate chromatin architecture in complex tissues. Cell.

[57] Carballo-Molina OA (2016). Semaphorin 3C released from a biocompatible hydrogel guides and promotes axonal growth of rodent and human dopaminergic neurons. Tissue Eng. Part A.

[58] Schindelin J (2012). Fiji: An open-source platform for biological-image analysis. Nat. Methods.

[59] Dunn KW, Kamocka MM, McDonald JH (2011). A practical guide to evaluating colocalization in biological microscopy. Am. J. Physiol. Cell Physiol..

[60] Buenrostro JD, Wu B, Chang HY, Greenleaf WJ (2015). ATAC-seq: A method for assaying chromatin accessibility genome-wide. Curr. Protoc. Mol. Biol..

[61] Martin M (2011). Cutadapt removes adapter sequences from high-throughput sequencing reads. EMBnet J..

[62] Bolger AM, Lohse M, Usadel B (2014). Trimmomatic: A flexible trimmer for illumina sequence data. Bioinformatics.

[63] Andrews, S. A quality control tool for high throughput sequence data. http://www.bioinformatics.babraham.ac.uk/projects/fastqc/. http://www.bioinformatics.babraham.ac.uk/projects/fastqc/.

[64] Langmead B, Salzberg SL (2012). Fast gapped-read alignment with Bowtie 2. Nat. Methods.

[65] Li H (2009). The sequence alignment/map format and SAMtools. Bioinformatics.

[66] Buenrostro JD, Giresi PG, Zaba LC, Chang HY, Greenleaf WJ (2013). Transposition of native chromatin for fast and sensitive epigenomic profiling of open chromatin, DNA-binding proteins and nucleosome position. Nat. Methods.

[67] Zhang Y (2008). Model-based analysis of ChIP-Seq (MACS). Genome Biol..

[68] Amemiya HM, Kundaje A, Boyle AP (2019). The ENCODE blacklist: Identification of problematic regions of the genome. Sci. Rep..

[69] Quinlan AR, Hall IM (2010). BEDTools: A flexible suite of utilities for comparing genomic features. Bioinformatics.

[70] Anders S, Pyl PT, Huber W (2015). HTSeq-A Python framework to work with high-throughput sequencing data. Bioinformatics.

[71] Love MI, Huber W, Anders S (2014). Moderated estimation of fold change and dispersion for RNA-seq data with DESeq2. Genome Biol..

[72] Heinz S (2010). Simple combinations of lineage-determining transcription factors prime cis-regulatory elements required for macrophage and B cell identities. Mol. Cell.

[73] Subramanian A (2005). Gene set enrichment analysis: A knowledge-based approach for interpreting genome-wide expression profiles. Proc. Natl. Acad. Sci. U. S. A..

[74] Kuleshov MV (2016). Enrichr: A comprehensive gene set enrichment analysis web server 2016 update. Nucleic Acids Res..

[75] Chen EY (2013). Enrichr: Interactive and collaborative HTML5 gene list enrichment analysis tool. BMC Bioinform..

[76] Cuevas-Diaz Duran R (2017). The systematic analysis of coding and long non-coding RNAs in the sub-chronic and chronic stages of spinal cord injury. Sci. Rep..

[77] Trapnell C (2010). Transcript assembly and quantification by RNA-Seq reveals unannotated transcripts and isoform switching during cell differentiation. Nat. Biotechnol..

