# Supplementary material for: Dynamic landscape of chromatin accessibility and transcriptomic changes during differentiation of human embryonic stem cells into dopaminergic neurons
Source: Sci Rep. 2021 Aug 20;11:16977. doi: 10.1038/s41598-021-96263-1 (PMC8379280; doi:10.1038/s41598-021-96263-1)
Supplement: Supplementary file 14 — Supplementary Information 11. [file 41598_2021_96263_MOESM14_ESM.docx]

**SUPPLEMENTARY INFORMATION**

**Dynamic landscape of chromatin accessibility and transcriptomic changes during differentiation of human embryonic stem cells into dopaminergic neurons**

César Meléndez-Ramírez ^1,2,6^, Raquel Cuevas-Díaz Durán ^3,6,*^, Tonatiuh Barrios-García ^3^, Mayela Giacomán-Lozano ^3^, Adolfo López-Ornelas ^1,2,4^, Jessica Herrera-Gamboa ^3^, Enrique Estudillo ^2^, Ernesto Soto-Reyes ^5^, Iván Velasco ^1,2,*^, Víctor Treviño ^3,*^

# **Supplementary Methods**

**Dopaminergic differentiation of hESCs**

The human embryonic stem cell line H9 (WA09)-GFP, which has constitutive expression of enhanced Green Fluorescent Protein (GPF), was used for all experiments ^1^. Prior to differentiation, cells were cultured in supplemented Knock Out (Gibco ®) medium, previously conditioned with mitotically inactivated mouse embryonic fibroblasts (MEFs) with fresh FGF-2 (10 ng/ml; Sigma), over a Matrigel (BD ®) matrix, until they reached 75% confluency. The floor-plate dopaminergic differentiation procedure was described previously ^2^ with minor modifications ^3^. The condition with conditioned medium before starting the dual SMAD inhibition, was considered day zero and cells pluripotent. Pharmacological inhibition of BMP and TGF-b receptors was made with LDN193189 (100 nM; Stemgent) and SB431542 (10 mM; Tocris), respectively. Wnt canonical pathway was stimulated by inhibiting GSK-3b with CHIR99021 (3 mM; Stemgent). Shh receptors were stimulated by SAG (1 mM; Sigma) and purmorphamine (2 mM; Stemgent). Recombinant human FGF-8 was added at 100 ng/ml (Peprotech). At day 14, cells were cultured in Neurobasal medium with B27 supplement and the morphological changes were evident; cells were positive in a high proportion to NESTIN, indicating that neural progenitors were predominant at this stage. The neuronal differentiation and survival were promoted by BDNF (20 ng/ml; Peprotech), ascorbic acid (0.2 mM; Sigma), GDNF (20 ng/ml; Peprotech), TGF-β3 (1 ng/ml; Peprotech), dibutyryl cAMP (0.5 mM; Sigma) and the Notch inhibitor DAPT (10 mM; Sigma) in the culture medium. At day 28, many cells presented neuronal morphology and expressed TH and FOXA2, indicating appropriate differentiation to dopamine neurons. The samples were analyzed at 0, 14 and 28 days.

**Immunostaining**

For immunofluorescence assays of hESCs, 150,000 feeder mitotically-inactivated MEFs on 24-well plates with coverslips treated with 0.5% porcine Gelatin and cultured in DMEM (Gibco ®) supplemented with 10% fetal bovine serum for 36 h. Then, 300,000 H9-GFP cells were plated per well over feeder cells and cultured for 2-3 days with Knock Out DMEM supplemented with Knock Out Serum Replacement (Gibco ®). Neural progenitors were re-seeded at days 13 or 21 of differentiation on 24-well plates with glass coverslips treated with Poly-L-ornithine (15 µg/ml), Fibronectin (1 µg/ml) and Laminin (5 µg/ml). Cells re-seeded at day 13 were fixed the next day and those re-seeded at day 21 continued the differentiation protocol and were fixed at day 28. For fixation, cell culture medium was removed, and cells were washed twice with PBS before being fixed with PFA 4% at 4°C for 20 minutes. After fixation, cells were washed twice with PBS.

For immunodetection, cells were permeabilized for 25 minutes with 0.5% Triton X-100 on PBS and later washed with PBS for 5 minutes. Non-specific detection was blocked with 10% goat serum in PBS for an hour at room temperature. Cells were then incubated overnight at 4°C with primary antibodies diluted in 10% goat serum in PBS with the following concentrations: CTCF rabbit (1:2000, Millipore 07729), OCT4 mouse (1:250, BD 611202), SOX2 rabbit (1:500 Millipore AB5603), TUJ1 rabbit (1:2000, Covance MMS435P-100), NESTIN rabbit (1:500, Covance 839801), FOXA2 rabbit (1:500, Millipore 07633) and TH mouse (1:1000, Pel-freez 401010). The primary antibodies solution was removed, followed by three 5-minute PBS washes. Then, cells were incubated for 2 h at room temperature with agitation with the following secondary antibodies diluted in 10% goat serum in PBS: Alexa Fluor 568 goat anti-mouse (1:600, Invitrogen) and Alexa Fluor 647 goat anti-rabbit (1:600, Jackson). The cells were then washed three times with PBS for 5 minutes and incubated with Hoechst 33258 (1 ng/ml) for nuclear labeling. After a single wash with PBS the samples were mounted on a glass slide using Aqua-Poly/Mount.

**Western Blot**

Western blots of CTCF and TH were performed according to standard protocol. Primary antibodies were used at the following concentrations: CTCF rabbit (1:2000, Millipore 07729), TH rabbit (1:2000), GAPDH mouse (1:5000), β-actin mouse (1:1000; Santa Cruz). The secondary antibodies were used at the following concentrations: goat anti-mouse IgG (1:10000; Santa Cruz) and goat anti-rabbit IgG (1:5000; Santa Cruz) both coupled to horseradish peroxidase. Luminata Forte (Merck®) was used for signal development and images were acquired using a gel imaging system. Images obtained were analyzed using the Gels tool from ImageJ software. CTCF signal was normalized according to the β-actin signal.

**Analysis of CTCF protein levels by immunofluorescence**

To analyze CTCF protein levels, photographs of CTCF- and Hoechst-stained cells were taken at days 0, 14 and 28. For each experiment, 100 nuclei were considered, with 3 biological replicates per condition. ROI manager tool from ImageJ software was used to obtain average intensity of normalized fluorescence.

**CTCF spatial dynamics analysis by immunofluorescence**

Confocal microscope was used to capture the images, adjusting the Z parameter to a minimum to obtain a thin section. Photographs were analyzed using ImageJ software. Colocalization of CTCF with Hoechst was determined by merging images and measuring magenta area (red + blue signal) by color threshold adjustment. The percentage of colocalization was obtained with respect to the total area of the nucleus.

**CTCF Statistical Analysis**

Statistical analysis was done using the GraphPad PRISM® 6.0 software. One-way ANOVA with *post hoc* Tukey tests were performed for CTCF expression by RNA-seq, fluorescence intensity comparison, spatial dynamic of CTCF, and CTCF protein level by *Western Blot*. An alpha value < 0.05 was considered significant.

**CTCF and H3K27me3 co-localization**

Cultures were fixed at D0, D14 and D28 and decorated with anti-CTCF antibodies (a kind gift from Dr. Félix Recillas-Targa) and anti-H3K27me3 antibodies (Abcam ab6002). Secondary antibodies coupled to Alexa 647 and 568 were used to detect the primary antibodies. Stack images were acquired in a Nikon A1R HD25 to generate 3D reconstructions presented as videos in Supplemental Information. Optical sections were used to measure the co-localization of CTCF and H3K27me3, by calculating the correlation coefficient, as reported ^4^.

**ATAC and RNA sequencing**

ATAC libraries of human ES cells induced into dopaminergic neurons were prepared for days 0, 14, and 28 following a previously published method ^5^. Briefly, 50,000 cells were detached from each induction plate at the previously defined time-points and washed with cold PBS. Next 50 μl of cold lysis buffer was added to the cell pellet. The Tn5-mediated tagmentation and adapter incorporation were performed with the Nextera DNA Library Prep Kit (Illumina) following manufacturer’s protocol at 37ºC for 30 min. The transposed DNA was purified using Qiagen MinElute PCR Purification Kit (Qiagen). To amplify DNA fragments, we used NEBNext High-Fidelity PCR Master Mix (New England Biolabs). The quality of the purified libraries was assessed by a Bioanalyzer High-Sensitivity DNA Analysis Kit (Agilent) and quantified using the KAPA Library Quant Kit for Illumina Sequencing Platforms (KAPA Biosystems). Paired-end 75 cycle sequencing reads were obtained on the Illumina HiSeq 2500 Sequencer.

Total RNA was extracted at dopaminergic induction days 0, 14, and 28 using TRIzol reagent (Invitrogen) following manufacturer’s instructions. RNA quality was determined using a Bioanalyzer (Agilent) and only samples with RNA integrity numbers (RINs) greater than 8 were used. Approximately 150–300 ng of total RNA was used to construct each RNA-Seq library. Poly-A selected RNA samples were used for library construction with Illumina TruSeq RNA Sample Prep Kit (Illumina). Samples were run on MiSeq and HiSeq 2500 Illumina sequencer using paired-end 75 cycle configuration. Approximately 80.4 million passed-filter reads were obtained encompassing 3 replicates of D0, 4 replicates of D14 and 3 replicates of D28 of differentiation. Similar gene expression patterns were found when we comparing our results with the gene expression levels previously reported by microarrays ^2^.

**ATAC-Seq pre-processing**

After removing adaptors using cutadapt ^6^ and trimmomatic ^7^ we obtained 36-126 bp paired-end ATAC-Seq reads for each time-point samples. The quality of trimmed sequencing datasets was verified using FASTQC ^8^. We followed the ENCODE ATAC-Seq pipeline (http://www.encodeproject.org/atac-seq/) to process ATAC-Seq samples. Paired-end reads were aligned to the human reference genome (GRCh38/hg38) using Bowtie2 with parameters ‘-X 2000 -k 5’ ^9^. Next, we used SAMTools ^10^ to remove unmapped reads, fragments with unmapped mates, non-primary alignment reads, and reads failing platform quality checks. Low quality reads (MAPQ < 30) and reads which mapped to mitochondrial DNA were also excluded. Optical and PCR duplicates were also removed using Picard tools (http://broadinstitute.github.io/picard). To accurately locate the center of each transposon-binding event, the remaining reads were offset by +4 and -5 for positive and negative strands respectively ^11^. To evaluate the expected periodicity of DNA winding around nucleosomes, we obtained insert size histograms using Picard tools (Fig.4a and Supplementary Fig. S1). ATAC-Seq peak regions were called for each sample using MACS2 with parameters --shift 75 --extsize 150 --nomodel --keep-dup all --call-summits ^12^. Resulting peaks which overlapped with ENCODE blacklisted regions were filtered out ^13^.

**Analysis of differential chromatin accessibility**

To generate a consensus set of unique peaks throughout all time-points, we merged peaks which were less than 100 bp apart both within and between samples. In total, we identified 464,783 consensus peaks. To find peak regions exclusive of specific time-points, we used BEDTools ^14^. For each sample we found the number of reads across each peak region using HTSeq ^15^. To assess the significant changes in chromatin accessibility between time-points, we used DESeq2 ^16^ with default parameters. Peak regions were considered differentially accessible in a sample with |log_2_(FC)| > 1.5 and p-value < 0.05. We performed pairwise comparisons to identify time-point differentially accessible peaks. Sequencing-depth normalized coverage plots (Figure 4D, Figure S2) and heatmaps were generated using deepTools2 ^17^. We used the Integrative Genomics Viewer (IGV) ^18^ to browse and compare peak regions among samples (Figures 5B,5C, and Figure S3).

**Genomic features of ATAC-Seq peaks**

We used HOMER annotatePeaks function ^19^ to associate ATAC-Seq peaks with the nearest genes according to GENCODE v29 human annotation file (https://www.gencodegenes.org/). Additionally, peaks were annotated to the following categories: intergenic, promoter-TSS, exon, intron, 5’ UTR, 3’ UTR, and TTS. Promoter-TSS was defined as the region 1 kb upstream and 100 bp downstream of the reference TSS as defined by the GENCODE gene annotation file. Similarly, transcription termination site (TTS) was defined as the region 100 bp upstream and 1 kb downstream of the reference TTS.

**Gene-set enrichment analysis**

Gene-set enrichment analysis were performed using a hypergeometric statistical test (phyper R function). The collections of annotated gene-sets including KEGG, REACTOME, and BIOCARTA canonical pathways as well as gene ontologies were downloaded from the Molecular Signatures Database (MSigDB) ^20^. Gene-sets used for gene-set enrichment analysis were combined with Wikipathways 2019 human database obtained from Enrichr ^21,22^. Gene-sets were considered significantly enriched with FDR < 0.05.

**Public Datasets**

For comparison purposes, we used the public human datasets indicated below. Datasets were converted to human hg38 genome assembly using UCSC liftOver tool ^23^. Additionally, we used GeneHancer database from GeneCards Suite v4.14 ^24^ for comparison of enhancer regions and predicted target genes.

| **Dataset type** | **Tissue or cell line** | **Accession number** |
| --- | --- | --- |
| CTCF ChIP-seq | H1 | GSM822297 |
| DNase | H9 | GSM878612 |
| H3K27ac | H9 | GSM605307 |
| H3K27me3 | H9 | GSM667622 |
| H3K4me1 | H9 | GSM667626 |
| H3K4me3 | H9 | GSM616128 |
| DNase | Neural progenitor cells | GSM878615 |
| H3K27ac | Neural progenitor cells | GSM818031 |
| H3K27me3 | Neural progenitor cells | GSM818032 |
| H3K27me3 | Neural progenitor cells | GSM772801 |
| H3K4me1 | Neural progenitor cells | GSM772808 |
| H3K4me3 | Neural progenitor cells | GSM772736 |
| H3K27me3 | Differentiated neuron | GSM772787 |
| H3K4me1 | Differentiated neuron | GSM772785 |
| H3K4me3 | Differentiated neuron | GSM772776 |
| CTCF ChIP-seq | Differentiated LUHMES | GSM2948735 |
| H3K27ac | Differentiated LUHMES | GSM2948731 |
| H3K27ac | Brain substantia nigra | GSM997258 |
| H3K27me3 | Brain substantia nigra | GSM669953 |
| H3K4me3 | Brain substantia nigra | GSM670038 |

**Transcription factor binding analysis**

To assess the potential regulatory function of TFs over differentially expressed protein-coding and lncRNA genes, we performed a motif search using position weight matrices of known and discovered motifs from the ENCODE TF ChIP-seq datasets ^25^. Gene sequences of promoter genomic regions defined by 1 kb upstream and 1 kb downstream of the reference TSS were obtained using the BSgenome R package ^26^. Genomic regions were scanned for motif occurrences using FIMO ^27^. TF binding motifs with FDR < 0.1 were considered significant. Similarly, the occurrence of TF binding sites in common and differentially accessible chromatin regions was determined using the DNA sequence of peak regions. The list of resulting TF binding motifs was further filtered to exclude motifs from TFs which were not differentially expressed. The most frequently occurring DE TFs were selected for a correlation analysis. The linear correlation between the normalized gene expression of selected TFs and DEGs was calculated using Pearson coefficient. Only correlations with FDR < 0.05 were considered significant.

**Gene expression analysis**

We removed adaptor sequences using cutadapt ^6^ and trimmomatic ^7^. We obtained 36-76 bp paired-end reads for each time-point samples. The quality of raw sequenced reads was verified using FASTQC ^8^. We used a previously described pipeline for read mapping, transcript assembly, and expression estimation ^28^. Reads were mapped to the human reference genome hg38 (https://www.gencodegenes.org/) using TopHat v2.1.1 ^29^ with default parameters. Assembly of mapped reads was performed with Cufflinks v2.2.1 ^30^ and values of Fragments Per Kilobase of transcript per million Mapped reads (FPKM) were calculated for all annotated genes and transcripts. A comprehensive gene annotation including protein-coding genes and lncRNAs was obtained from GENCODE v29 (https://www.gencodegenes.org/). We used HTSeq ^15^ to calculate read counts for annotated genes and transcripts. Next, we adjusted batch effect (Fig. S2) and performed pairwise comparison of read counts using DESeq2 ^16^. Genes were labelled as differentially expressed (DEG) if at least one of the replicates in the comparison had FPKM $\geq$ 1, and normalized count FC > 4 with an FDR < 0.05. Only DEGs were used in downstream analysis.

Temporal gene expression profiles of DEGs were obtained using hierarchical clustering. In this unsupervised clustering method, we implemented Ward’s linkage algorithm using the Euclidean distance matrix of log2 transformed FPKM values of DEGs. DEGs in each cluster were used for gene-set enrichment analysis as previously described. A list of DEGs is included in Supplementary Table S2.

**Analysis of DE lncRNAs**

To analyze the differences between the number of lncRNAs and protein-coding genes found within the top ranked genes (10, 20, 30, 40, and 50) we performed a hypergeometric test using the phyper function implemented in R. Figure 3a shows the number of genes belonging to each category. An asterisk highlights the comparisons in which the *p*-value of the number of lncRNAs is smaller than the *p*-value corresponding to protein-coding genes. Number of DEGs and *p*-values are included in Supplementary Table S4.

GENCODE annotation included gene and transcript biotypes. LncRNA biotypes included antisense, lincRNA, processed transcript, sense intronic, sense overlapping, and TEC (to be experimentally confirmed) categories. To determine the statistical significance of the number of DE lncRNAs obtained in each group, we performed a hypergeometric test using the phyper R function. We obtained *p*-values from the number of lncRNAs in each category of all pairwise comparisons. We plotted the number of DE lncRNAs per category (Fig. 3b) and included asterisks to indicate the degree of significance. Number of annotated and DE lncRNAs as well as *p*-values are included in Supplementary Table S4.

# **Supplementary Figures**


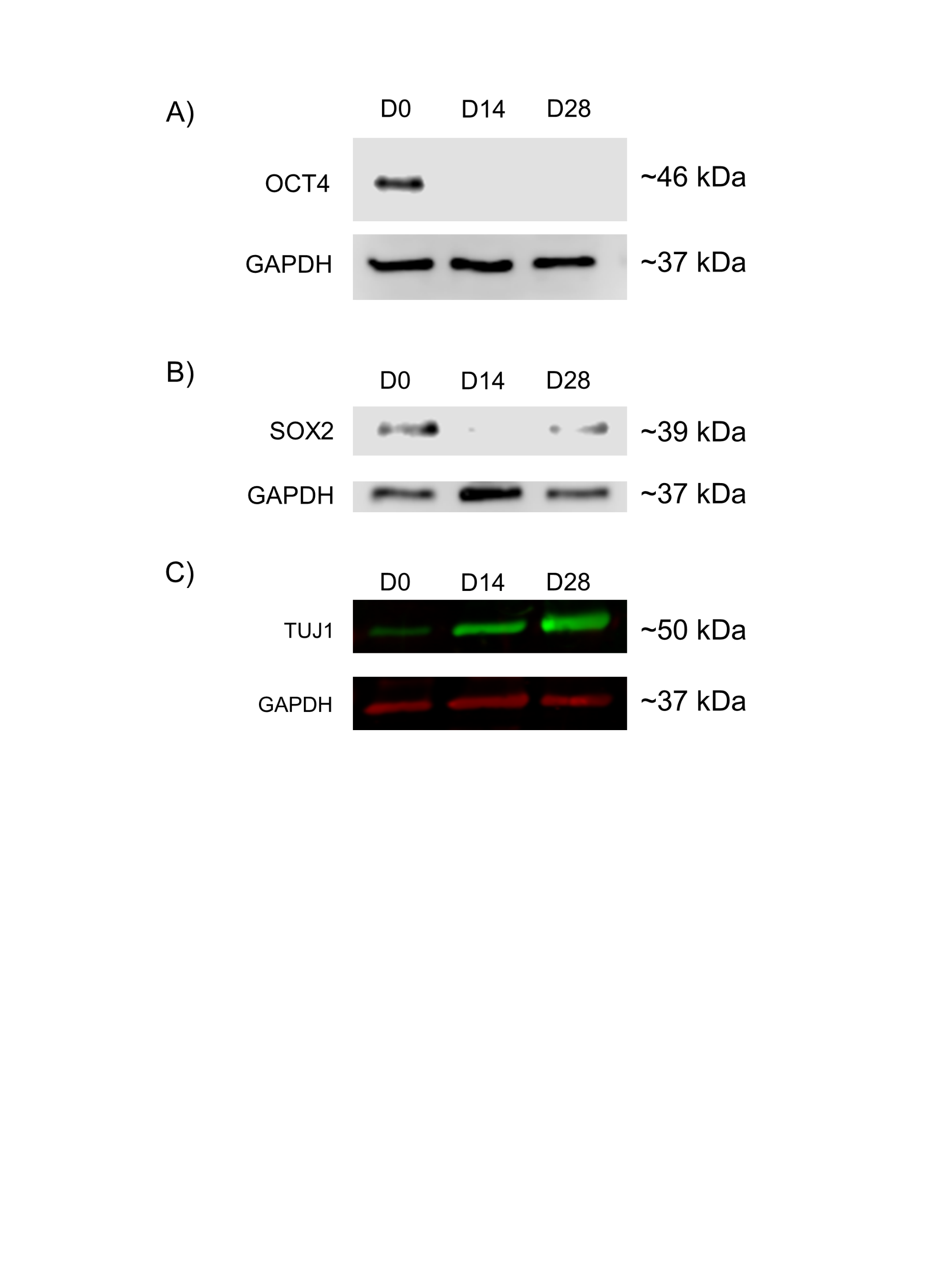


**Figure S1. Protein dynamics during dopaminergic differentiation.**

A) Representative experiment of OCT4 (~46 kDa) detection by immunoblot, during dopaminergic induction. GAPDH was used as a loading control (~37 kDa). B) Representative experiment of SOX2 (~39 kDa) detection by immunoblot, during dopaminergic induction. GAPDH was used as a loading control (~37 kDa). C) Representative experiment of TUJ1 (~50 kDa) detection by immunoblot, during dopaminergic induction. GAPDH was used as a loading control (~37 kDa).

**Figure S2. Principal component analysis plot depicting batch effect adjustment and similarity among replicates of the same time-point.**

Batch effect was modeled and adjusted using DESeq2 ^16^. Plots were built using the top 1000 most variable genes. Percentages indicate the amount of variability explained by each principal component.

**Figure S3. Hierarchical clustering analysis displaying temporal expression profiles of known pluripotency, neural, and dopaminergic marker genes.** **Related to Fig. 2.**

Scale indicates row z-scores of log2 transformed FPKM gene expression values.

**Figure S4. Coverage plots depicting chromatin accessibility in diverse scenarios. Related to Fig. 4.**

**(**A) Chromatin accessibility in promoter regions of differentially expressed genes (DEGs) belonging to distinct transcriptional profile clusters. Numbers represent the cluster number as defined in Fig. 2. (B) Chromatin accessibility in promoter regions of DEGs classified as protein-coding (blue lines) or lncRNA (red lines). Numbers represent the cluster number as defined in Fig. 2. (C) Chromatin accessibility in 500 randomly selected non-expressed gene promoter regions.

**
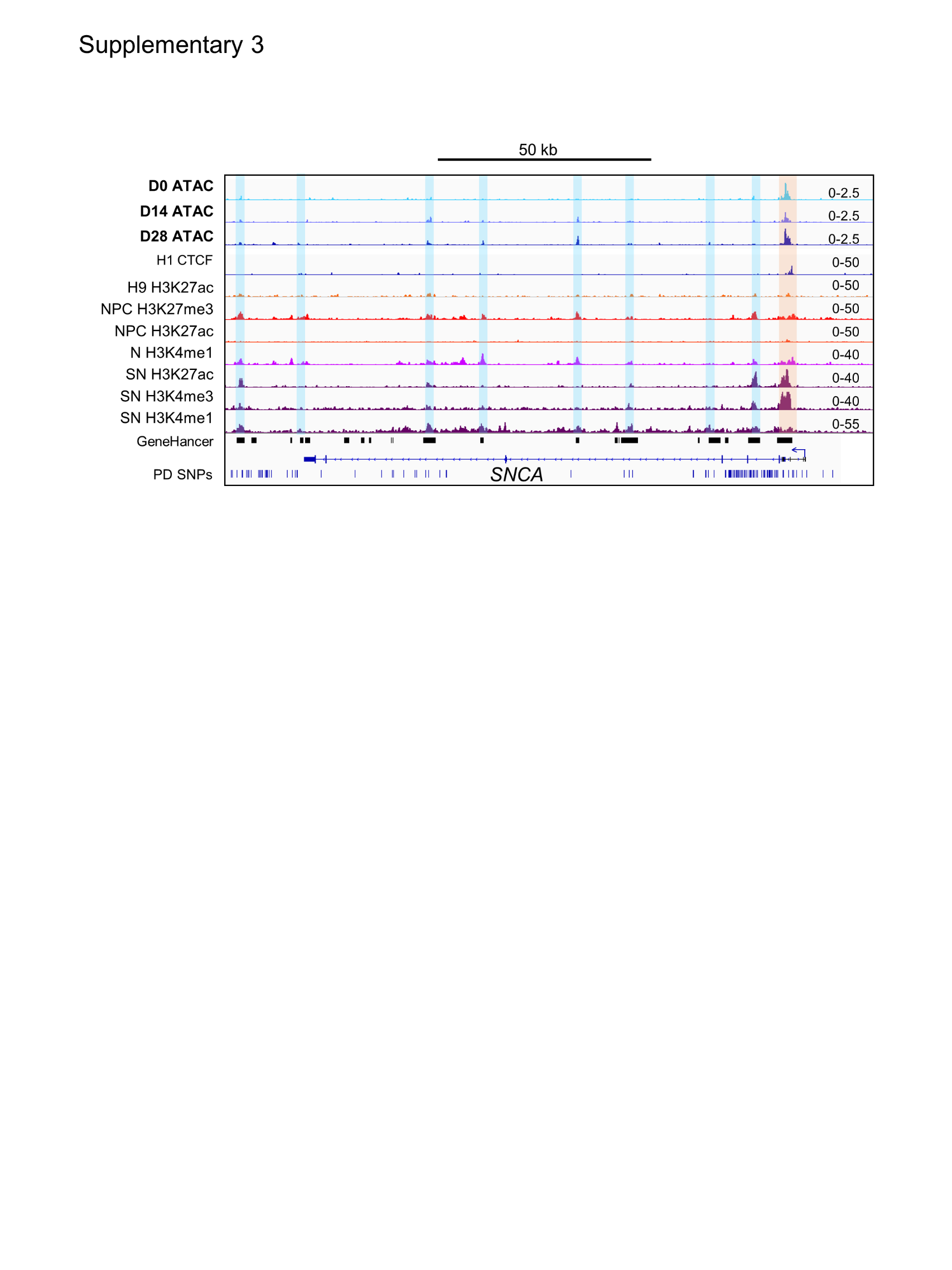
**

**Figure S5. Changes in chromatin accessibility and gene expression in SNCA, a well know PD risk *locus*.**

(A) ATAC-Seq derived chromatin accessibility and RNA-Seq gene expression tracks in the *SNCA* locus. ENCODE tracks of H1 CTCF, H9 H3K27ac, ESC-derived Neural precursors (NPC) H3K27me3, ESC-derived neurons (N) H3K4me1, *Substantia nigra* (SN) H3K4me1, H3K4me3 and H3K4me1 are included for comparison. Tracks of predicted and known enhancers from GeneHancer database and PD SNPs ^31^ are also included. Orange highlights *SNCA* promoter region. Blue zones mark putative enhancers. Track data ranges are shown in the right. Scale represents 50 kb.

**Figure S6. Histograms depicting gene expression correlation in all induction time-points between selected highly enriched TFs (CTCF, HEY1, SP8 and BHLHE40) and DEGs**. **Related to Fig. 6.**

Dotted red lines represent FDR < 0.05 of Pearson correlation coefficient. Blue plots indicate the corresponding TF mean gene expression at D0, D14 and D28.


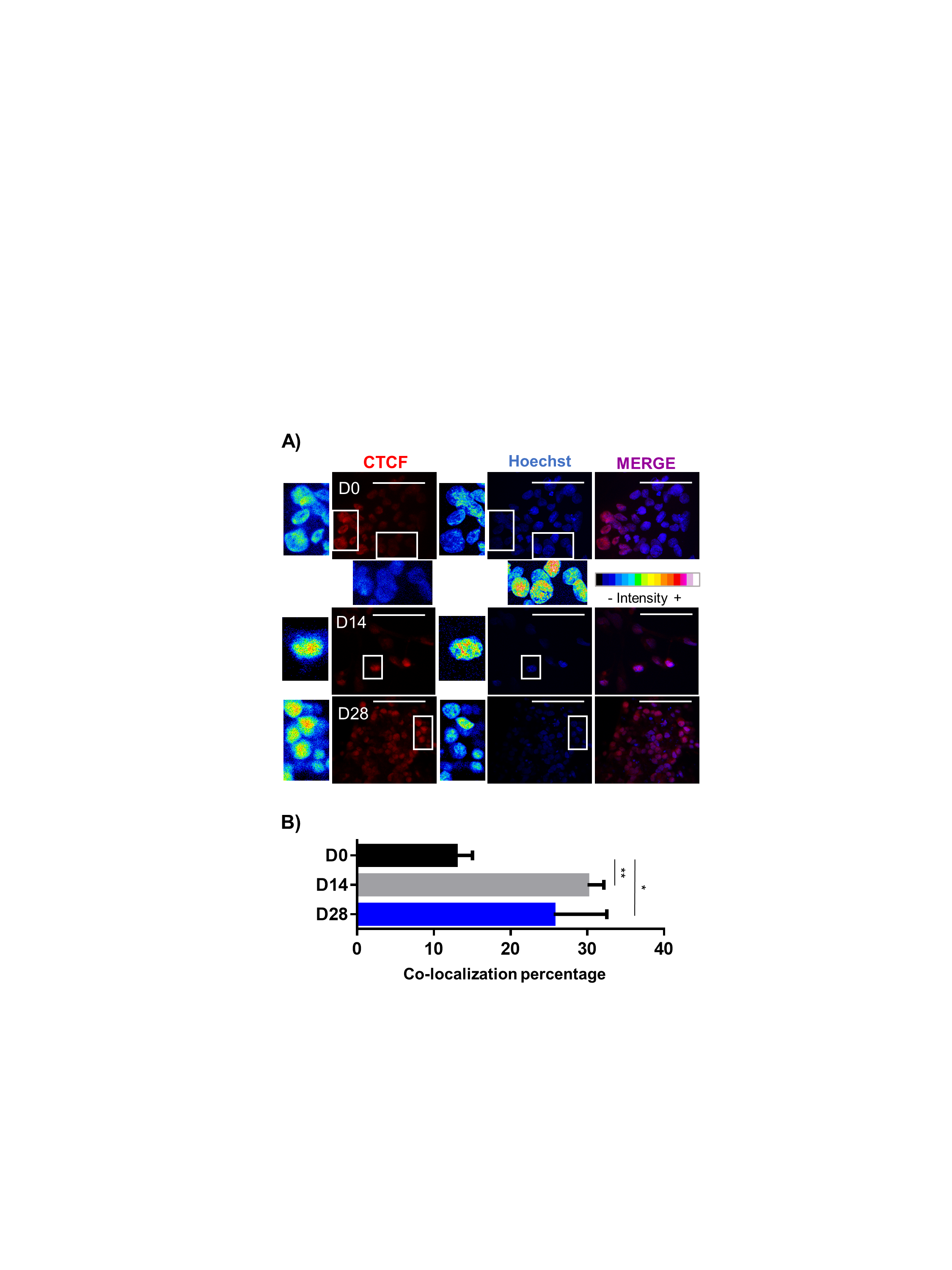


**Figure S7. CTCF colocalization with Hoechst.**

A) Confocal microscope fluorescence images showing immunostaining of CTCF and Hoechst labeling through dopaminergic differentiation. The regions marked with rectangles from a representative picture were filtered with a 16 color heatmap to show the intensity of each signal. Magenta represents merged image and the bars represent100μm. B) Percentage of colocalization of CTCF and dense Hoechst regions during the differentiation quantified from 3 independent experiments by confocal microscopy. *p< 0.05;**p< 0.01.


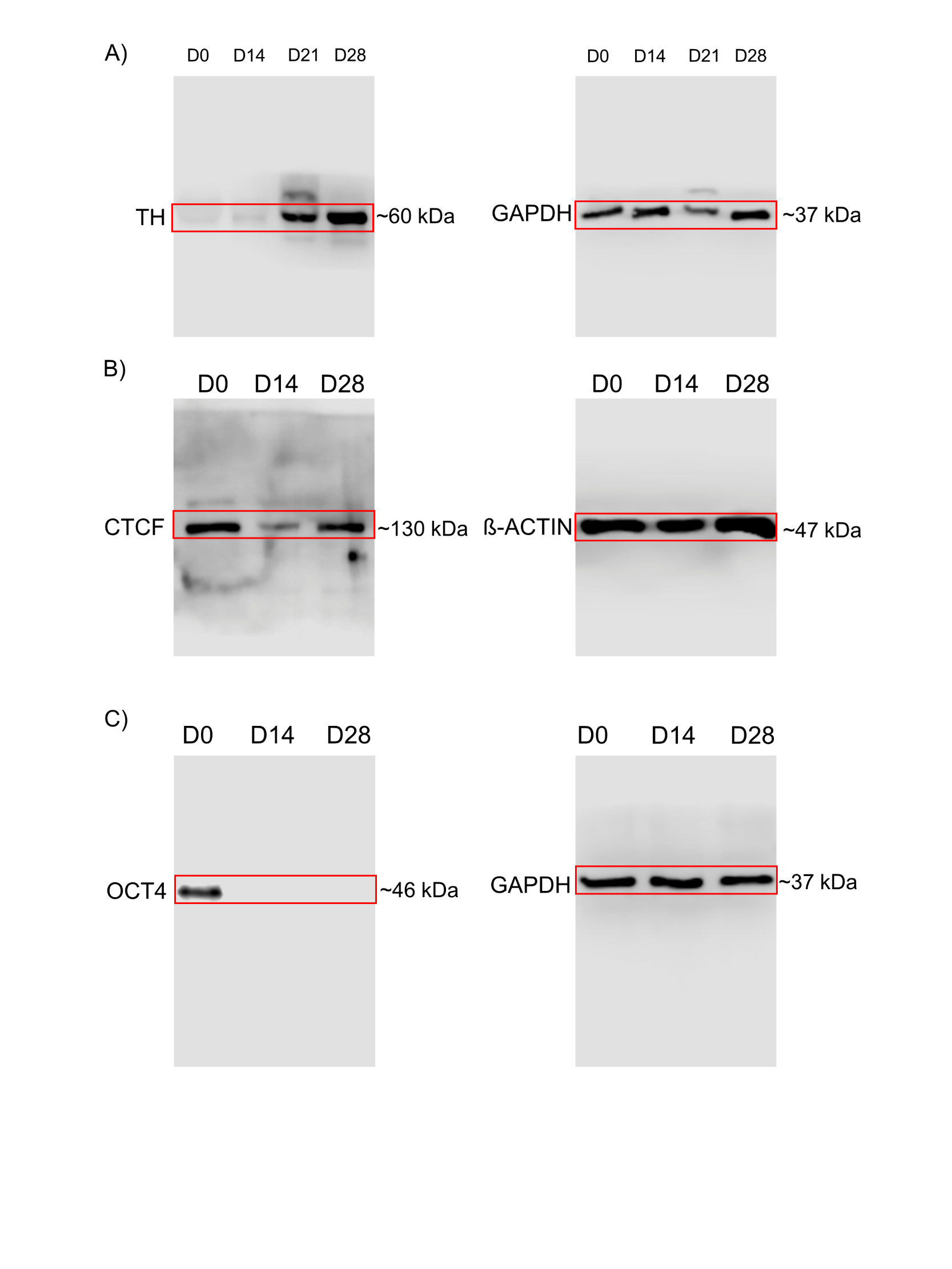


**
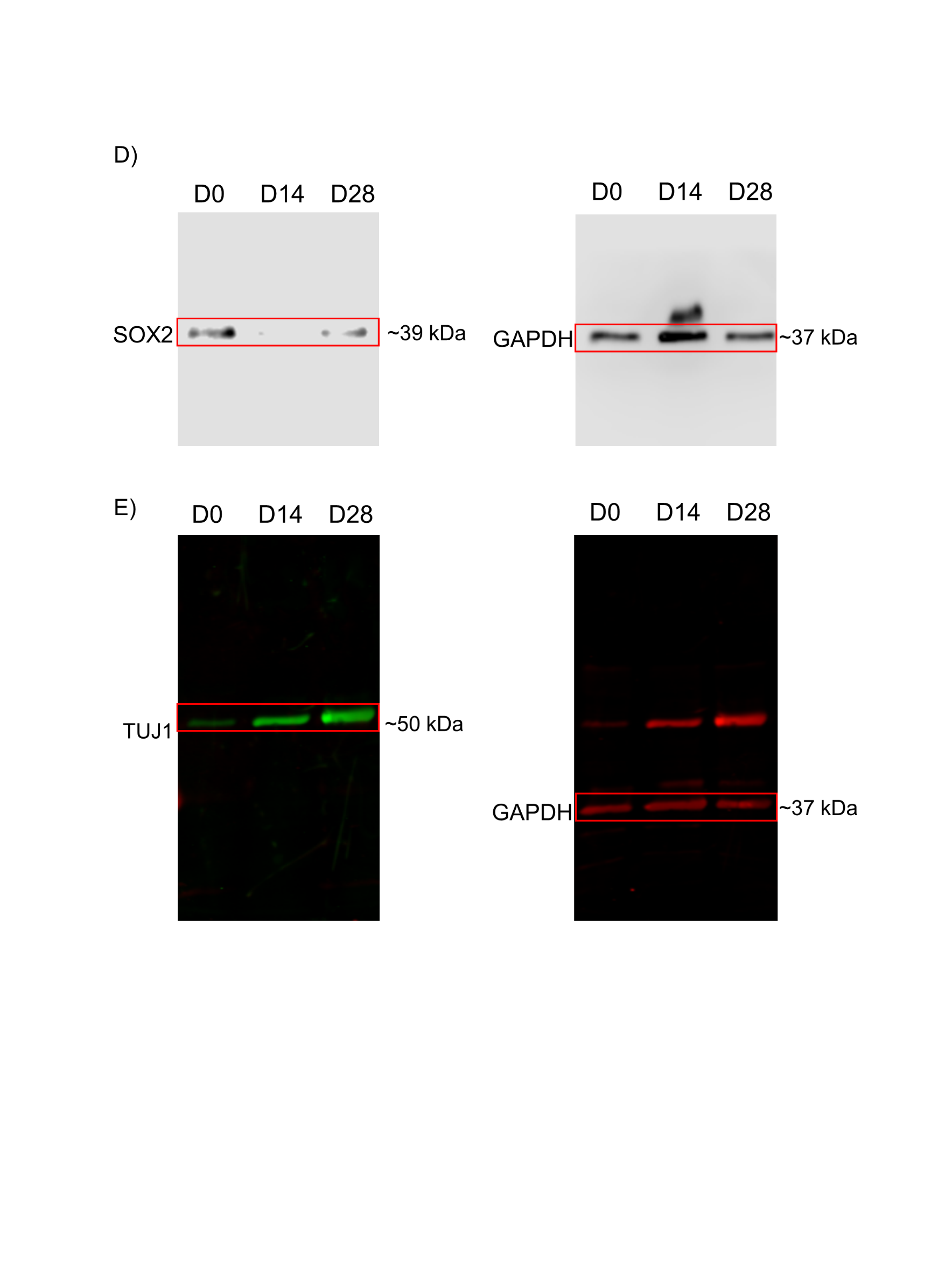
**

**Figure S8. Uncropped Western blot gels.**

A) Uncropped view of the representative gel, presented in Fig. 1, to determine TH (~130 kDa) protein levels with respect to GAPDH (~37 kDa). B) Uncropped view of the representative gel, shown in Fig. 7, to determine CTCF (~130 kDa) protein levels with respect to ß-ACTIN (~47 kDa). C) Uncropped view of the representative gels included in Fig. S1 to show OCT4 (~46 kDa), SOX2 (~39 kDa) and TUJ1 (~46 kDa) protein levels, with respect to GAPDH (~37 kDa). Note that TUJ1 bands are still detectable in GAPDH immunoblotting, since both antibodies (anti-TUJ1 and anti-GAPDH) were raised in mouse.

# **Supplementary References**

1. Domingo-Reines, J. *et al.* Hoxa9 and EGFP reporter expression in human Embryonic Stem Cells (hESC) as useful tools for studying human development. *Stem Cell Res.* **25**, 286–290 (2017).

2. Kriks, S. *et al.* Dopamine neurons derived from human ES cells efficiently engraft in animal models of Parkinson’s disease. *Nature* **480**, 547–51 (2011).

3. Carballo-Molina, O. A. *et al.* Semaphorin 3C released from a biocompatible hydrogel guides and promotes axonal growth of rodent and human dopaminergic neurons. *Tissue Eng. Part A* **22**, 850–861 (2016).

4. Dunn, K. W., Kamocka, M. M. & McDonald, J. H. A practical guide to evaluating colocalization in biological microscopy. *Am. J. Physiol. - Cell Physiol.* **300**, C723-42 (2011).

5. Buenrostro, J. D., Wu, B., Chang, H. Y. & Greenleaf, W. J. ATAC-seq: A method for assaying chromatin accessibility genome-wide. *Curr. Protoc. Mol. Biol.* **109**, 21.29.1-21.29.9 (2015).

6. Martin, M. Cutadapt removes adapter sequences from high-throughput sequencing reads. *EMBnet.journal* **17**, 10–12 (2011).

7. Bolger, A. M., Lohse, M. & Usadel, B. Trimmomatic: A flexible trimmer for Illumina sequence data. *Bioinformatics* **30**, 2114–2120 (2014).

8. Andrews, S. A quality control tool for high throughput sequence data. http://www.bioinformatics.babraham.ac.uk/projects/fastqc/. http://www.bioinformatics.babraham.ac.uk/projects/fastqc/.

9. Langmead, B. & Salzberg, S. L. Fast gapped-read alignment with Bowtie 2. *Nat. Methods* **9**, 357–359 (2012).

10. Li, H. *et al.* The Sequence Alignment/Map Format and SAMtools. *Bioinformatics* **25**, 2078–9 (2009).

11. Buenrostro, J. D., Giresi, P. G., Zaba, L. C., Chang, H. Y. & Greenleaf, W. J. Transposition of native chromatin for fast and sensitive epigenomic profiling of open chromatin, DNA-binding proteins and nucleosome position. *Nat. Methods* **10**, 1213–8 (2013).

12. Zhang, Y. *et al.* Model-based analysis of ChIP-Seq (MACS). *Genome Biol.* **9**, R137 (2008).

13. Amemiya, H. M., Kundaje, A. & Boyle, A. P. The ENCODE Blacklist: Identification of Problematic Regions of the Genome. *Sci. Rep.* **9**, 9354 (2019).

14. Quinlan, A. R. & Hall, I. M. BEDTools: A flexible suite of utilities for comparing genomic features. *Bioinformatics* **26**, 841–842 (2010).

15. Anders, S., Pyl, P. T. & Huber, W. HTSeq-A Python framework to work with high-throughput sequencing data. *Bioinformatics* **31**, 166–169 (2015).

16. Love, M. I., Huber, W. & Anders, S. Moderated estimation of fold change and dispersion for RNA-seq data with DESeq2. *Genome Biol.* **15**, 550 (2014).

17. Ramírez, F. *et al.* deepTools2: a next generation web server for deep-sequencing data analysis. *Nucleic Acids Res.* **44**, W160-5 (2016).

18. Robinson, J. T. *et al.* Integrative genomics viewer. *Nat. Biotechnol.* **29**, 24–26 (2011).

19. Heinz, S. *et al.* Simple Combinations of Lineage-Determining Transcription Factors Prime cis-Regulatory Elements Required for Macrophage and B Cell Identities. *Mol. Cell* **38**, 576–89 (2010).

20. Subramanian, A. *et al.* Gene set enrichment analysis: A knowledge-based approach for interpreting genome-wide expression profiles. *Proc. Natl. Acad. Sci. U. S. A.* **102**, 15545–15550 (2005).

21. Kuleshov, M. V. *et al.* Enrichr: a comprehensive gene set enrichment analysis web server 2016 update. *Nucleic Acids Res.* **44**, W90-7 (2016).

22. Chen, E. Y. *et al.* Enrichr: Interactive and collaborative HTML5 gene list enrichment analysis tool. *BMC Bioinformatics* **14**, 128 (2013).

23. Meyer, L. R. *et al.* The UCSC Genome Browser database: Extensions and updates 2013. *Nucleic Acids Res.* **41**, D64–D69 (2013).

24. Fishilevich, S. *et al.* GeneHancer: genome-wide integration of enhancers and target genes in GeneCards. *Database (Oxford).* **2017**, bax028 (2017).

25. Kheradpour, P. & Kellis, M. Systematic discovery and characterization of regulatory motifs in ENCODE TF binding experiments. *Nucleic Acids Res.* **42**, 2976–2987 (2014).

26. Pagès, H. BSgenome: Software infrastructure for efficient representation of full genomes and their SNPs. R package version 1.56.0. (2020).

27. Grant, C. E., Bailey, T. L. & Noble, W. S. FIMO: Scanning for occurrences of a given motif. *Bioinformatics* **27**, 1017–1018 (2011).

28. Cuevas-Diaz Duran, R. *et al.* The systematic analysis of coding and long non-coding RNAs in the sub-chronic and chronic stages of spinal cord injury. *Sci. Rep.* **7**, 41008 (2017).

29. Kim, D. *et al.* TopHat2: Accurate alignment of transcriptomes in the presence of insertions, deletions and gene fusions. *Genome Biol.* **14**, R36 (2013).

30. Trapnell, C. *et al.* Transcript assembly and quantification by RNA-Seq reveals unannotated transcripts and isoform switching during cell differentiation. *Nat. Biotechnol.* **28**, 511–515 (2010).

31. Nalls, M. A. *et al.* Identification of novel risk loci, causal insights, and heritable risk for Parkinson’s disease: a meta-analysis of genome-wide association studies. *Lancet Neurol.* **18**, 1091–1102 (2019).

**Supplementary Tables legends.**

**Table S1. Protein-coding and lncRNA gene expression levels (FPKM), normalized counts, and batch adjusted normalized counts.**

The column ‘type’ is used to differentiate protein-coding genes from lncRNAs. The column ‘specific type’ corresponds to the specific gene biotype according to GENCODE annotation file. Data in these Tables were used in downstream analysis.

**Table S2. Differential expression analysis results obtained from temporal pairwise comparisons (D0 vs D14, D14 vs D28, D0 vs D28).**

Analysis was performed with DESeq2 using the raw counts for all annotated genes. Genes were considered differentially expressed with FC > 4, FDR < 0.05 and FPKM > 1. A heatmap of DE genes is depicted in Fig. 2c.

**Table S3. Hierarchical cluster membership of DE protein-coding genes and lncRNAs.** Related to heatmap in Fig. 2c. Additionally, the gene-set enrichment results obtained using genes in each cluster is included in tabs: gsea_cluster_1, gsea_cluster_2, gsea_cluster_3, gsea_cluster_4, gsea_cluster_5, and gsea_cluster_6. GSEA results include gene-set, p-value, number of genes, FDR, and log10(FDR). A heatmap of DE genes is depicted in Fig. 2c.

**Table S4. Counts and hypergeometric tests used to determine the statistical significance of categories in Fig. 3a and 3b.**

To compare the number genes belonging to lncRNAs against those in protein-coding gene categories within the top 10, 20, 30, 40, and 50 DE genes in each pairwise comparison (Fig. 3a), we performed a hypergeometric statistical test. A similar analysis was performed for the number of DE genes in each lncRNA category (Fig. 3b). We used a hypergeometric test to determine the statistical significance of the number of DE lncRNAs in each category, and each pairwise comparison, considering the number of annotated lncRNAs. The number of annotated and DE lncRNAs per category as well as the *p*-values obtained from each statistical test are included.

**Table S5. Gene expression Pearson correlations between DE lncRNAs and target protein-coding genes.**

We compiled a list of 96 pairs of DE lncRNAs and their known protein-coding target genes. We obtained the Pearson correlation of normalized gene expression between all pairs of DE lncRNAs and target protein-coding genes in samples from all time points (D0, D14, and D28). We obtained 54 pairs of genes with significant correlation (*p*-value < 0.05). Tabs for each pairwise comparison are included and contain the list of DE lncRNAs and target protein-coding gene in each time point. Related to Fig. 3c.

**Table S6. Significantly enriched gene-sets found using differentially accessible peak regions in each induction time point (D0, D14, and D28).**

Peak regions were considered differentially accessible with log2 FC > 1.5 and p-value < 0.05. Gene-sets were considered significantly enriched with FDR < 0.05. Selected enriched gene-sets are depicted in Fig. 4e and 4f.

**Table S7. Overlap of PD-associated SNPs with consensus and time-point specific open chromatin regions.**

We performed hypergeometric tests to determine if the numbers of genome-wide peaks within specific genomic regions (intronic, intergenic, promoter, TSS, exonic, 5’/3’) overlapping PD-related SNPs ^28^ were statistically significant (*p*-value < 0.05).

**Table S8.** **Transcription factor binding sites found in promoter regions (1 kb upstream and 1 kb downstream) of DE protein-coding genes, lncRNAs, and genes in clusters.**

For motif search we used FIMO (FDR < 0.1) with position weight matrices downloaded from ENCODE. Motifs were further filtered to include only those belonging to differentially expressed TFs. Related to Fig. 6a and 6b.

**Table S9.** **Percent of TFBS in promoters and enhancers derived from consensus open chromatin regions**. Most frequent TFs are depicted in Fig. 6c.

**Table S10. Mean FPKM values of CTCF isoforms in different time points.**

**Video S1. 3D reconstruction of the co-localization of CTCF with the facultative heterochromatin mark H3K27me3 at D0.**

**Video S2. 3D reconstruction of the co-localization of CTCF with the facultative heterochromatin mark H3K27me3 at D14.**

**Video S3. 3D reconstruction of the co-localization of CTCF with the facultative heterochromatin mark H3K27me3 at D28.**
